# Supplementary material for: Why women choose to give birth at home: a situational analysis from urban slums of Delhi
Source: BMJ Open. 2014 May 22;4(5):e004401. doi: 10.1136/bmjopen-2013-004401 (PMC4039791; doi:10.1136/bmjopen-2013-004401)
Supplement: Author's manuscript [file bmjopen-2013-004401.draft_revisions.pdf]

Why do women deliver at home? A situational analysis from urban slums of Delhi

Journal:	*BMJ Open*
Manuscript ID:	bmjopen-2013-004401
Article Type:	Research
Date Submitted by the Author:	04-Nov-2013

Complete List of Authors:	Devasenapathy, Niveditha; Indian Institute of Public Health-Delhi, Public Health Foundation of India,<br>George, Mathew; Public Health Foundation of India, Indian Institute of Public Health-Delhi<br>Ghosh-Jerath, Suparna; Public Health Foundation of India, Indian Institute of Public Health-Delhi<br>Singh, Archna; All India Institute of Medical Sciences, Department of Biochemistry<br>Negandhi, Himanshu; Public Health Foundation of India, Indian Institute of Public Health-Delhi<br>Alagh, Gursimran; Public Health Foundation of India, Indian Institute of Public Health-Delhi<br>Shankar, Anuraj; Harvard School of Public Health, Department of Nutrition<br>Zodpey, Sanjay; Public Health Foundation of India, Indian Institute of Public Health-Delhi

**Primary Subject Heading**:	Public health
Secondary Subject Heading:	Health services research
Keywords:	PUBLIC HEALTH, QUALITATIVE RESEARCH, Quality in health care < HEALTH SERVICES ADMINISTRATION & MANAGEMENT

For peer review only - http://bmjopen.bmj.com/site/about/guidelines.xhtml

Why do women deliver at home? A situational analysis from urban slums of Delhi

Niveditha Devasenapathy[1], MBBS, M.Sc, Mathew Sunil George[1], MPH, Suparna Ghosh Jerath[1], PhD, Archna Singh[2], PhD, Himanshu Negandhi[1], MD, Gursimran Alagh B.Sc[1], Anuraj H Shankar[3], D.Sc, Sanjay Zodpey[1] MD, PhD

[1] Indian Institute of Public Health, Delhi, Public Health Foundation of India, Gurgaon, India,
[2]Department of Biochemistry, All India Institute of Medical Sciences, New Delhi, India
[3]Department of Nutrition, Harvard School of Public Health, Boston, Massachusetts, USA

Address for correspondence

Dr Niveditha Devasenapathy MBBS, MSc
Assistant Professor
Indian Institute of Public Health- Delhi
Plot No. 34, Sector 44, Institutional Area, Gurgaon 122002, India
Phone: 91-124-4722922, Fax : 91-124-4722901
email:niveditha@iiphd.org

Key Words: Reproductive Health, Access to Health Care, Health Promotion, Needs Assessment, Maternal Health

Word count: 3596

For peer review only

For peer review only - http://bmjopen.bmj.com/site/about/guidelines.xhtml

ABSTRACT

Background: Institutional delivery is an important strategy towards attaining MDG-5. Rapid growth of low-income and migrant populations in urban settings in India and other countries has placed additional demands on government programs to enhance utilization of institutional care. Better understanding of the barriers to institutional deliveries among urban poor is urgently needed to enhance program impact in India, and elsewhere.

Objectives: To measure prevalence of home deliveries in Delhi slums and identify the reasons for home delivery.

Study design: Cross-sectional survey using quantitative and qualitative methods

Methods: A house-to-house survey was conducted of all households in three slum-clusters in north-east Delhi (n=32,034 individuals). Data on deliveries and socio-demographic characteristics were collected using structured questionnaires (n=6092 households). Detailed information on pregnancy and postnatal care was obtained from women who had delivered in the last 3 months (n=160). Focus group discussions and in-depth interviews were conducted with stakeholders from the community and health-care facilities. Risk-factors for home delivery were examined using random effects logistic regression.

Results: Of 824 women who delivered in the previous year, 53%[95%CI 49.7-56.6] had delivered at home. In adjusted analyses, multiparity, low literacy and migrant status were independently predictive of home delivery. Fear of hospitals (36%), comfort of home (20.7%) and lack of social support for child-care (12.2%) emerged as reasons for home delivery.

For peer review only - http://bmjopen.bmj.com/site/about/guidelines.xhtml

Conclusion: Home deliveries are frequent among the urban poor. In addition to current financial initiatives for institutional delivery, raising community awareness and improvements in client services and family support are key modifiable factors identified in this study. These findings should inform the design of strategies to promote institutional delivery.

Article Summary

Strengths and Limitations of the study

- This survey covered a large number of households (n= 6092) households living in 3 urban poor settlements of Delhi and both qualitative and quantitative methods were used to capture reasons for home delivery.
- Though the slum cluster was not a random sample from all the slum clusters in Delhi they were representative of the urban poor settlements.
- Concurrent health facility assessment was not done which would have helped to understand the supply side issues.

For peer review only - http://bmjopen.bmj.com/site/about/guidelines.xhtml

BACKGROUND

Institutional delivery is currently a key global strategy to reduce maternal and newborn mortality. Many countries, including India, have established incentive programs and policies to enhance institutional deliveries. However, the rapid growth of low-income urban populations presents unique challenges to these programs, and more targeted efforts may be required to improve institutional delivery rates in low-income urban settings. India currently accounts for about a fifth of all maternal deaths worldwide.[1] The maternal mortality ratio (MMR) was 200 as of 2010.[2] With approximately one third of the population currently living in urban areas, and growing to nearly one half by 2030, a large proportion of maternal and newborn deaths occur amongst the urban poor. The MDG report has flagged the slow progress of India in reducing child mortality and improving maternal health.[3] Current trends suggest that India is unlikely to achieve MDG 4 and 5 by 2015.[4] At least four antenatal visits to hospital and deliveries conducted by skilled birth attendants are identified as key interventions to reduce maternal mortality. The latest WHO statistics show that in India only half of expectant mothers complete four Antenatal care (ANC) visits and deliver their baby in the presence of a skilled birth attendant.[5]

The choice of place of delivery has been driven by tradition, accessibility and economics. In India, MMR is high in states with high prevalence of home deliveries.[6] Janani Suraksha Yojana (JSY) a safe motherhood intervention under the National Rural Health Mission (NRHM)was implemented in India in 2005 with the objective of reducing maternal and neonatal mortality by promoting institutional delivery among poor pregnant women.[7] An evaluation of this

For peer review only - http://bmjopen.bmj.com/site/about/guidelines.xhtml

conditional cash transfer scheme in 2007-08 showed an increase in ANC visits and institutional deliveries.[8] However, this increase in institutional deliveries has not translated to reduction in MMR probably due to unaddressed issues of non-financial access barriers and sub-optimal ANC and postnatal care.[9] Currently 50% of India population are living in cities. Delhi is one of the most densely populated cities in the world. Delhi attracts nearly 500,000 migrants every year most of who mostly settle down in urban poor habitations.

According to the National Family Health Survey (NFHS 3) survey conducted in 2005-06, only 44 % of deliveries were institutional among the urban poor of Delhi as compared to the urban average of 67.5%.[10] The District Level Household and Facility Survey (2007-08) survey showed that overall, 71% of pregnant women had at least 3 ANC visits. While 68% of deliveries were institutional in the city as a whole, only 38% institutional deliveries were reported in slum areas.[11] A governmental initiative aimed at correcting this inequity is the National Urban Health Mission (NUHM) which makes essential primary health care services available to the urban poor.[12],[13] The success of this mission will depend on identifying and targeting interventions directed towards the most vulnerable. One of the aims of this study is to determine the prevalence of home delivery among women living in urban poor settlements in Delhi and identify reasons behind their choice to deliver at home.

For peer review only

METHODS

This analysis is part of the formative phase and situational analysis for the ANCHUL (Ante Natal and Child Health care in Urban Slums, CTRI/2011/091/000095) study which is an implementation research project aimed to develop, implement and evaluate the effectiveness of an intervention package delivered through an urban community healthcare worker (UCHW). ANCHUL aims to increase access to institutional deliveries and improve maternal, neonatal, child healthcare (MNCH) practices in urban slums of Delhi. The objective of this formative study was to conduct an in-depth situational analysis on utilization and quality of MNCH care using quantitative and qualitative methods. The information obtained from this study will guide the development of the community based intervention package to be delivered by the UCHW as part of the ANCHUL trial.

Study Setting

Of the 16.7 million people living in Delhi, 52% reside in poor habitations.[14] The north-east district of Delhi contributes to 11% of the total population with 44 registered slums.[15] This district has the highest home delivery rates.[11] We conducted a rapid survey in 17 slum clusters to obtain information on number of households, water supply, sanitation, presence of schools, healthcare facility, and distance from nearest government hospital. The clusters were then stratified into two categories of vulnerability based on the above characteristics. We randomly chose three vulnerable slum clusters namely Buland Masjid (BM), CPJ and Chanderpuri (CP*)* for the purpose of this situational analysis. These slums had metalled roads within and had access to maternal child health care dispensaries within a distance of five kms.

For peer review only - http://bmjopen.bmj.com/site/about/guidelines.xhtml

The study protocol was approved by Health Ministry Screening Committee of the Government of India, institutional ethics committees of the Public Health Foundation of India, All India Institute of Medical Sciences, and Harvard School of Public Health.

Data collection

Quantitative survey

After lane mapping the clusters, all households were included in the survey. We identified pregnant women (in their 2nd and 3rd trimesters), recently delivered women (RDW, i.e., those who had delivered in the last 3 months) and households with under-5 children. The purpose of the survey was explained to a household member above 18 years of age and all questionnaires were administered after obtaining informed consent by trained field interviewers. All survey tools were in local language and were piloted and modified for content and clarity. Information on family details, socio-demographic status, place of delivery (in women who had delivered in past 1 year), and information on any maternal and child deaths within households in past 1 year, was obtained using paper forms. All refusals and non-responses were documented. We re-visited the households of 160 RDW and collected detailed information about ANC, delivery, immediate post-natal period, new born care practices and diet of the mother. Data were checked for completion before entering into a validated database (Microsoft access 2010) with inbuilt range and internal consistency checks. Information from RDW was validated by double data entry.

Qualitative data

The categories of respondents in *table 1* were identified as relevant for data collection in this study. Households were informed that focus group discussions (FGDs) would be held in the

For peer review only - http://bmjopen.bmj.com/site/about/guidelines.xhtml

community and a general invitation was given. Permissions were sought from local community and religious leaders. Local public and private health care providers were approached and permissions were sought for in-depth interviews (IDI). The FGD and IDI guides were piloted to refine the topic guides to enable them to generate data that was relevant to the study objectives. The main topics that were explored in the FGDs and interviews are shown in *table 1*. The venue for data collection was agreed upon based on the respondent's convenience. One interviewer facilitated the discussions while a second took notes. Based on responses from the community, health care facilities and traditional birth attendants who served the locality were identified and approached. Written informed consent was obtained from all participants before the FGDs and IDIs, which were digitally voice-recorded.

Sample size justification

For estimating the number of households to be interviewed, institutional delivery was considered the key outcome variable. Assuming prevalence of institutional deliveries as 33% in urban slums of Delhi[13], we needed to interview 780 women who delivered in past one year to obtain current prevalence estimates with 10% relative precision. Assuming a crude birth rate of 25/1000 (national average is 21/1000), a population of 30,000 was to be covered to identify at least 750 deliveries that happened in past one year.

For peer review only - http://bmjopen.bmj.com/site/about/guidelines.xhtml

Table 1: Themes covered for qualitative data

Category of participants	Method of data collection	Key themes covered
**Community**<br>• Pregnant women (n= 5)<br>• Recently delivered women (n=6)<br>• Mother of under 5 children (n=6)<br>• Mother in laws (n=5)<br>• Husbands (n=4)	Focus Group Discussions(FGDs)<br><br>Venue: Schools, NGO, Madrassa(religious place) and anganwadi centres*	• Health and nutritional status<br>• Cultural practices for nutrition during pregnancy<br>• Care seeking behaviour during pregnancy<br>• Barriers to accessing care during pregnancy<br>• Quality of care experienced in various health care settings (public and private)
**Health Care Providers**<br>• Public Health system (n=6)<br>• Private (n=5)<br>• Others (n=4) (AWW, TBAs)	In depth interviews<br>Venue: Clinics of health care providers or homes of key informants	• Care seeking pattern among the community during pregnancy<br>• Challenges to improving maternal and child health among urban poor<br>• Feasibility of proposed intervention
**ANC Clinic attendees** (n=9)	Exit interviews (pre and post ANC check-ups)<br>Venue: Clinics	• Experience of care during ANC visit<br>• Satisfaction levels of the individual about care

AWW: Anganwadi workers, TBA: Traditional birth attendants

*The word Anganwadi means "courtyard shelter" in Hindi. They were started by the Indian government in 1975 as part of the Integrated Child Development Services program to combat child hunger and malnutrition.

Data analysis

Quantitative data

Data were analysed using Stata 11 (Stata Corporation, College Station, TX). Descriptive statistics were used to provide a cluster, household and individual level profiles of the study population. Household survey data were analysed accounting for clustering at the slum level to control for both inter and intra-cluster variance. We used principal component analysis to compute household Socioeconomic Scales (SES). Dwelling characteristics, household income and household assets were included in this composite scale.[16] We used mutli-variable random

For peer review only - http://bmjopen.bmj.com/site/about/guidelines.xhtml

effects logistic regression to estimate the association of demographic variables with home delivery. Crude and adjusted odds ratios were calculated with 95% confidence intervals. For data from RDWs, Pearson chi-squared was used for categorical variables and t-tests for comparison of continuous variables.

Qualitative data

Verbatim transcripts were prepared in a standardized format that included basic demographic information of the participants and the interviewer's own observations within one week of conduct of IDI/FGD. Transcripts were uploaded to a software Atlas ti 6.1 (Scientific Software Development, City West, Berlin) and coded line-by-line using detailed themes and sub-themes that emerged from the data. After an initial round of coding with a representative sample of transcripts, the list of codes that were generated was reviewed in order to develop a structured code list which was then applied to the remaining transcripts. Illustrative quotations from the transcripts have been included in the results.

RESULTS

Of the 6348 households in the three defined clusters, 6092 (96%) households were interviewed between December 2011 and March 2012, covering a total population of 32,034. Nine households refused to participate and 247 households did not respond (locked houses) (figure 1). A total of 25 FGDs and 13 in-depth interviews were conducted in January and February 2012. The number of respondents in each FGD ranged from 7-12 members.

For peer review only - http://bmjopen.bmj.com/site/about/guidelines.xhtml

For peer review only

Population and cluster characteristics

The adult male to female ratio was 1000:825. Fifty eight percent of the population were migrants from Uttar Pradesh (73%) and Bihar (16%). Eighty percent of the population were living in the same locality for > 5 years. Of the total population, women in reproductive age (15-49 years) accounted for 25%, and 16.6% were under-5 children. The area was served by one referral hospital situated within a distance of 5kms, two outpatient dispensaries, 17 private clinics (registered and unregistered) and one laboratory within the clusters. The areas also have access to two referral hospitals situated at distance of about 10 kms.

For pe

Household characteristics

The median family size was 5 (IQR 4, 7) predominantly living as nuclear families (79.4%) and 63% of houses were self-owned. The head of the household (HOH) was the one considered as the decision maker but was not necessarily the primary wage earner of the family. Fifty nine percent of HOHs were illiterate and were unskilled labourers. Ration card, Below Poverty Line cards (BPL) and Rashtriya Swasthya Bima Yojna (RSBY) cards that are needed for claiming government run health schemes were possessed only by 50%, 31% and 24% of households respectively. Majority of the households lived in single-roomed concrete houses and with cemented or tiled flooring. A detailed socio-demographic profile of the study population is presented in *Table 2*. The household characteristics of the subsample of RDW and the overall population in the study area were similar indicating that our subsample households were representative of this area. Fifteen maternal deaths, 21 still-births and 41 under-5 child deaths were reported for the previous year. Of the total child deaths, 22 were in the neonatal period.

For peer review only - http://bmjopen.bmj.com/site/about/guidelines.xhtml

Figure 1: Quantitative survey sampling

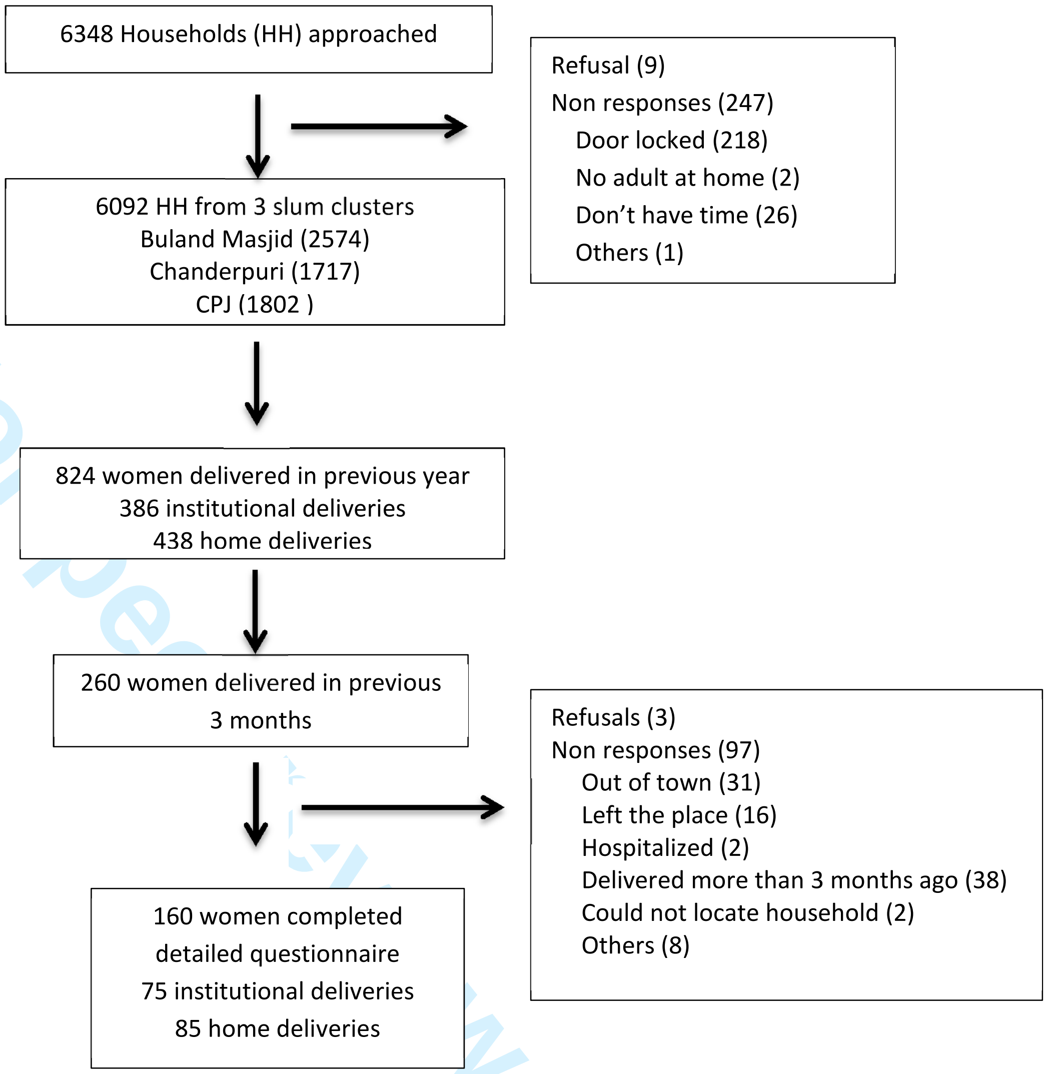

For peer review only

For peer review only - http://bmjopen.bmj.com/site/about/guidelines.xhtml

Table 2: Characteristics of households in the study area and households of women who delivered in previous 3 months

Demographic characteristics	House to House survey (n=6092 HH from 3 clusters	Recently Delivered Women (n=160)
Median HH size (IQR)	5(4,7)	5 (4,7)
Family type (%)		
Nuclear	4834(79.4)	115 (71.9)
Joint	944(15.5)	43 (26.9)
Extended	313 (5.1)	2 (1.3)
Spoken language (%)		
Hindi	5328 (87.5)	145 (90.6)
Urdu	624(10.2)	15 (9.4)
Others	140(3.2)	-
Religion (%)		
Hindu	1822(29.9)	42 (26.3)
Muslim	2475 (69.6)	118 (73.8)
Others	33(0.5)	-
Caste category (%)		
General	2546 (41.8)	77 (48.1)
Other Backward Caste	2553 (41.9)	58 (36.3)
Scheduled caste/Scheduled Tribe	932 (15.3)	22 (13.8)
Do not want to answer	4 (0.1)	-
Do not know	57 (0.9)	3 (1.9)
Illiterate women in reproductive age group (%)	(n= 8056) 4122(51.2)	85 (53.1)
Literacy level of HOH (%)		
Illiterate	3561(58.5)	93 (58.1)
Literate but no formal education	196(3.2)	4 (2.5)
Schooling	2129(36.4)	62 (38.8)
College	115(1.89)	1 (0.6)
Occupation of HOH (%)		
Unskilled	2805 (46.3)	77 (48.4)
Skilled	1378 (22.7)	36 (22.6)
Office work	867 (14.3)	19 (12)
Professional	55 (0.91)	-
Not working	955 (15.76)	27 (17)
Median HH income in INR (IQR)	4000(3000-6500)	4000 (3000-7000)
Median HH income in USD (IQR)	76.2(57.1- 123.8)	76.2 (57.1-133.5)
Own house (%)	3829 (62.9)	101 (63.1)
Ration card (%)		
Don’t have	2994 (49.2)	91 (56.9)
White	1173 (19.3)	22 (13.8)
Yellow	1196 (19.6)	28 (17.5)
Pink	686 (11.3)	18 (11.3)
Do not want to answer	43 (0.7)	1 (0.6)
BPL card (%)	1903 (31.2)	47 (29.4)

For peer review only - http://bmjopen.bmj.com/site/about/guidelines.xhtml

RSBY card (%)	1461 (24)	36 (22.5)
% of HH who are staying in the current locality in years		
< 1	660 (10.8)	13 (8.1)
1-2	238 (3.9)	11 (6.9)
3-5	330 (5.4)	12 (7.5)
>5	4864 (79.8)	124 (77.5)
% belonging to Delhi	3572(57.49)	93 (58.1)
% of HH who migrated but living in Delhi		
< 1 year	261 (7.5)	2 (2.2)
1-2	123 (3.5)	3 (3.2)
3-5 years	195 (5.6)	2 (2.2)
>5 years	2920 (83.5)	86 (92.5)
**Living conditions**		
Concrete houses (%)	4826 (79.2)	125 (78.1)
Cemented floor (%)	5829 (95.7)	153 (95.6)
Houses with only one room	4978 (81.7)	124 (77.5)
% of HH with separate kitchen	1168 (19.2)	33 (20.6)
Fuel used for cooking (%)		
Gas	3511 (57.6)	101 (63.1)
Kerosene	1831 (30.1)	44 (27.5)
Firewood	572 (9.4)	13 (8.1)
Electricity	92 (1.5)	2 (1.2)
Do not cook at home	86 (1.4)	-
Public source of drinking water (%) (handpump/ tanker/bore)	5102 (83.6)	132 (82.5)
Defecation facilities (%)		
Toilet within house	4571 (75.1)	125 (78.1)
Community/shared	1242 (20.4)	30 (18.8)
Defecate in open	278 (4.6)	5 (3.1)
Socioeconomic categories* (%)		
Lowest	1.976(32.45)	53 (33.1)
Middle	2,077 (34.11)	53 (33.1)
Highest	2,036 (33.44)	54 (33.8)
Assets (%) of HH in possession of		
Television	4350 (71.4)	112 (70)
Refrigerator	1557 (25.6)	46 (28.8)
Washing machine	1054 (17.3)	29 (18.1)
Mobile phone	4403 (72.3)	115 (71.9)
Distance of HH from nearest Maternal Child Health care centre		
<5 km	4882 (80.1)	125 (78.1)
5-10 km	1550 (18.9)	29 (18.1)
>10 km	60 (1)	6 (3.8)

HOH: Head of Houshold, BPL: Below Poverty Line card, RSBY: Rashtriya Swasthya Bhima Yojna(Health insurance scheme)*The scale is a composite of house type, floor, house ownership, separate kitchen, TV, Refrigerator, Mobile phone, washing machine, total HH income, Number of rooms by principal component analysis

For peer review only - http://bmjopen.bmj.com/site/about/guidelines.xhtml

Place of delivery

Of the 824 women who gave birth in the previous year, 438 delivered at home [53.1%, 95%CI (49.1 - 56.6)] and of the remaining 386, 340 (88%) chose to give birth at a public hospital. Among the women who delivered in the previous 3 months (n=160), a similar proportion [53.1 %, 95% CI (45.0- 61.0)] delivered at home. Only 16.2% (12) of these mothers' availed cash incentive through JSY scheme *(table 3)*. Thirty six (48%) went to hospital due to initiation of labour pains, 32% due to development of complications, and 6.7% reported the reason that they had crossed expected date of delivery. The individual who was most influential in the decision for delivering at a hospital was most often (48%) the women herself, followed by the husband (18.7%) or mother-in-law (17.3%). Irrespective of the place of delivery only 15% of these households were visited by an UCHW within 48hrs of delivery and only 30% of women visited a health care facility after delivery. Most (92%) were satisfied with the services provided at the hospital during delivery. Compared to women who delivered at a hospital, women who delivered at home were more likely to be multiparous, likely to avail ANC in a public hospital and visit a facility during the post-partum period *(table 3).*

Predictors of home delivery

Among the 824 women who delivered in the previous year, the following demographic characteristics were significantly associated with delivering at home: Living in rented house, low SES, low literacy of HOH, HOH being an unskilled labourer, migrants and multi-parity. Multi-parity [OR 1.96, 95% CI (1.44, 2.69)], literacy status of HOH [OR 0.71, 95%CI (0.53 0.97)] and migrant status [1.46, 95% CI (1.08, 1.97)] remained strong independent predictors of home delivery in multivariable analysis *(Table 4)*.

For peer review only - http://bmjopen.bmj.com/site/about/guidelines.xhtml

Table 3: Information of ANC and births obtained from recently delivered women

Characteristics*	Home deliveries (n=85)	Institutional deliveries (n=75)	p value
Mean age	24.7(4.31)	24.9(4.43)	0.79
>18yrs of age at marriage	72 (84.7)	64(85.3)	0.912
Mean family size	5.5(2.4)	5.5(2.3)	0.937
First child	15 (17.7)	26 (34.7)	**0.014**
Some ANC care	63 (74.1)	72 (96)	**<0.0001**
ANC at public hospital	41 (65)	61(84.7)	**< 0.0001**
ANC in first trimester	21 (33.33)	31 (43.1)	0.498
Some health problem during pregnancy	11 (17.5)	13 (18)	0.92
Satisfaction with ANC	46 (74.6)	65 (90.3)	0.046
Planned deliveries	73 (86)	66 (90.4)	0.38
Delivery conducted by Doctor/ Nurse	15 (17.7)	74 (98.7)	**<0.0001**
Home visitation by a community health worker	14 (16.5)	11 (14.7)	0.75
Post-delivery visit to hospital	3 (3.53)	16 (21.33)	**0.001**

*All continuous variables are expressed as mean and one standard deviation; all proportions are expressed as percentages

Table 4: Predictors of home delivery

Characteristics (n=824)<br>438: Home delivery<br>386: Institutional delivery	p value (ignoring clustering)	Crude OR (95% CI), p value (accounted for clustering)	Adjusted* OR (95% CI), p value (LR test)
Buland Masjid<br>CPJ<br>Chaderpuri	<0.001	1 **<0.001**<br>0.42(0.30, 0.59)<br>0.69 (0.49, 96)	
Birth order second and above	<0.001	2.12 (1.57, 2.87) **<0.001**	1.96 (1.44, 2.69) **<0.001**
Lower SES<br>Middle SES<br>Highest SES	0.033	1<br>0.90 (0.64, 1.26) **0.011**<br>0.70 (0.50 0.98)	0.96 (0.66, 1.406) 0.68<br>0.91 (0.60, 1.38)
Joint families	0.057	0.78 (0.57, 1.05) 0.102	
Non-Muslims	0.004	0.77 (0.54, 1.1) 0.15	0.78 (0.54, 1.14) 0.21
Schooling of HOH	0.007	0.74 (0.55, 0.99) **0.04**	0.71 (0.53 0.97) **0.031**
Not working<br>Elementary job<br>Skilled job	0.024	1 **0.063**<br>1.63(1.08, 2.45)<br>1.42 (0.92, 2.17)	1 0.2<br>1.54 (1, 2.38)<br>1.40 (0.89, 2.21)
Own the house	0.004	0.76 (0.56, 1.02) **0.07**	0.94 (0.65, 1.34**)** **0.72**
Ration card possession	0.037	0.80 (0.60, 1.07) 0.14	
Not belonging to Delhi	<0.001	1.61(1.21, 2.15) **0.001**	1.46 (1.08, 1.97) **0.013**

*Adjusted for house ownership, SES, literacy of HOH, Occupation of HOH, belonging to Delhi and birth order

For peer review only

Reasons for delivering at home

The majority of home deliveries were pre-planned and 75% of these women had availed some ANC at a facility. Eighty two percent of the home deliveries were conducted by a traditional birth attendant (*Dai*). The results from the quantitative and qualitative data, showed a high level of concordance for the reasons for choosing to deliver at home. Four major themes emerged as barriers to institutional delivery. Illustrative quotations from the transcripts are presented in *table 5*.

Table 5: Illustrative quotations from the transcripts for reasons for delivering at home

Fear and Embarrassment<br><br>*"They prefer home deliveries as in many cases the doctor does not behave well with them. As soon as they enter, they are separated from their families. The doctor does not communicate to the relative if there is any complication. They stay for 1-2 days in the labour room; the relatives are outside they do not know what is happening. It is very scary for them. Also because of all these reasons, they will come only if it is life threatening. Even then they might prefer to go to someone who is local, who is more patient friendly, private practitioner who are non-judgemental who behave properly. They give more one to one care. They might not be qualified but it is more natural and human."* ***Senior gynaecologist, Public health facility***<br><br>*"I'd prefer to have the baby at home. If you're in hospital, they don't even attempt to try for a natural birth. What's the use of having an operation if you can have a child the normal way? We get the check-ups done there, but we end up having the delivery at home".* ***Pregnant woman***<br><br>*"If you tell them to put you in a closed room to get a check - up, they tell you to just lie down and get it done right there. It's humiliating; you can't help but feel embarrassed. And if you don't feel embarrassed, other people around you will. They tell you that if you feel ashamed, go to a private hospital. It's a matter of dignity. There are men walking around as well, if a man catches a glimpse, it can create trouble at home".-****Pregnant woman***
Prior experience with hospitals<br><br>*"I had gone recently with my sister to a hospital……when I went there to deliver my baby, they just kept telling me 'keep pushing, keep pushing…" I got so scared I just left. I've had three children at home; I can manage a fourth the same way".* ***Recently delivered woman***
**Other children**<br><br>*"I have small children. If I have to go to the hospital, I have to lock the house and take the children along.*

For peer review only - http://bmjopen.bmj.com/site/about/guidelines.xhtml

Then if she delivers in the hospital, she may be admitted for at least 2 or 3 days depending on the situation. Even if it is a normal delivery it is at least a 2 day stay. How do I manage in such situations? To avoid all this one hopes that if all is well it is better to deliver at home itself. We can all be at home and kids need not have to go anywhere. I can also go for work.” ***Husband of pregnant woman***
**Opportunity costs**<br><br>*“Most of them earn daily wages, so they do not want to come to the hospital. They feel one day will go so they will lose their pay also...So the delivery can be at home if baby is okay. Male member does not want to involve himself in all these things, either if there is an elder woman in the house or neighbourhood who conducts the delivery...Even at the hospital they do not want to stay they say that they have to go to work, or how will they earn for tomorrow’s food because they are working on daily wages..”* ***Senior gynaecologist, public health facility***

Fear and embarrassment

Fear associated with hospitals was reported as the most important reason for delivering at home during FGDs and IDIs and was also reported as the key reason (35%) among the 85 RDW surveyed. Women feared interventions during delivery, particularly caesarean sections. Lack of privacy during deliveries and unfamiliar surroundings of hospitals as opposed to the ‘safe environment’ at their homes were also reasons for choosing to deliver at home.

Prior experience with hospitals

Prior experience of self, friend, neighbour or a family member played an important role in the choice to deliver at home or in hospital. Positive experiences reinforced the message that hospitals were a safe and welcoming place as opposed to negative experiences (such as, perceived improper care and rude behaviour of hospital staff). The health care providers interviewed indicated that high patient load at hospitals lead to lack of individual attention and inadequate care.

For peer review only - http://bmjopen.bmj.com/site/about/guidelines.xhtml

Domestic responsibilities

Being in an unfamiliar neighbourhood, the absence of extended family to help with childcare and traditional lack of involvement of men in childcare made women reluctant to leave children at home and get admitted to hospital. In the survey, 10% of those who delivered at home cited lack of help with childcare as a reason.

Opportunity costs

Though most services at the hospital were provided free of cost, opportunity costs in the form of lost wages for the earning member, cost of food for the family and travel, dissuaded some women from delivering in hospital. Although only 6% mentioned this as a reason in our survey, this emerged as an important factor in qualitative analysis.

DISCUSSION

Our study showed a high prevalence of home deliveries conducted by *dais* among the urban poor of north-east district of Delhi. Fear of surgical procedures, unfamiliarity with hospital surroundings, lack of help for childcare and loss of wages, were some of the reasons that drove women to choose home delivery. Other predictors of home delivery were low literacy, higher parity and migrant status. Concordance between results derived from qualitative and quantitative data lends greater credibility to these findings.

The prevalence and reasons for home deliveries in our study was similar to that found in most other urban surveys[10 11 17-23] from India *(Table 6).* In the Mumbai slum study[22], the

For peer review only - http://bmjopen.bmj.com/site/about/guidelines.xhtml

For peer review only

prevalence varied from 6-16% across 48 slum clusters. Tradition was the most important reason behind home delivery in this study. Apart from the predictors that we identified, poor housing, lack of water supply and hazardous location were associated with home deliveries in the Mumbai study, indicating that apart from individual and household level factors, the type of neighbourhoods also played a role. One of the limitations of our study is that we could not evaluate cluster level predictors of home delivery as we included only three clusters in this study.

Migrant status was one of the important determinants of home delivery in our study. Analyses using NFHS data, Singh et al[24] reported that urban poor migrants were at highest risk of unsafe delivery practices in contrast to non-poor, non-migrants who were at least risk. In our study sample almost 60% of the households were migrants from neighbouring states and most had been living in Delhi for more than 5 years. In spite of this, these households were less likely to possess ration, BPL or RSBY cards which are required for availing entitlements to healthcare.

The dissatisfaction among the care seekers that we observed could be attributed to the overburdening of referral hospitals. Antenatal care is provided at dispensaries, maternal and child care centres (MCH), secondary level and referral hospitals. However, MCH centres cater to deliveries of multigravida only, and all primigravida are referred to the secondary level or referral hospitals leading to increased patient load at these centres. Initiatives to decentralize care to reduce burden on referral hospitals by upgrading the MCH centres has been rather slow. It might be possible that other supply-side issues could have also contributed to this level of dissatisfaction, but due to delay in obtaining permissions, we were unable to conduct facility assessment to identify the potential causes.

For peer review only - http://bmjopen.bmj.com/site/about/guidelines.xhtml

Table 6: Prevalence and reasons for home delivery from urban surveys in India since 2000

Author, year of publication (ref)	Study area and target population/ Study design	Sample size and prevalence of home deliveries	Reasons for home deliveries
Rahi et al, 2006[17]	One Urban slum in Delhi, Births recorded during April-June 2005, cross sectional survey	n= 82 births<br><br>Home deliveries= 56.1%	Not reported
Agarwal[18] et al, 2007	One urban slum in Delhi, Women who delivered last 1 year, Cross sectional survey	n= 82<br>Home deliveries = 31.8%	Lack of awareness for need for check-up (27%)<br>Lack of knowledge about service availability (17%)<br>Long waiting time (22%)<br>None to accompany (15%)<br>Finance (12%)<br>Fear of hospitals (7%)<br>Family objections (2%)
DLHS Fact sheet (2007-8)[9]	Delhi state in 2008 using multistage stratified probability sampling	n= 9689 households<br>Rural = 42.6%<br>Urban = 29.9%<br>Total = 30.8%	Not reported
Thind et al, 2008[15]	NFHS survey data from Maharashtra , cross sectional survey	n=1510 recent births<br>Home deliveries(overall) = 37%<br>Only Urban = 15.3%	Predisposing factors Religion(Hindu), multiple births and caste
Agarwal S et al , 2010[19]	11 slums of Indore, MP, Cross sectional survey of mothers of infants(2004-06)	n= 312<br>Home deliveries = 56.4%	Not reported
Das S, 2011[20]	Mumbai slums from 6 municipal wards, survelllance study (2005-2007)	n= 10,754 births<br>Home deliveries =10%	Customary(28%) , No time to reach hospital(13%), no body to go along(8%), Fear(7%)
Dasgupta et al, 2006[16]	Rural and urban clusters in West Bengal from Birbhum district. Cross sectional survey, women who delivered in the last one year	n= 320<br>Home deliveries (rural and urban combined) =51.88%	Not reported
Khan Z et al, 2009 [21]	Periurban area of Aligarh, Uttar Pradesh	n=92 mother of infants<br>Home deliveries = 60%	Tradition (42%)<br>Related to economics(31%)
Hazarika, 2009[8]	NFHS- 3 Delhi data, Cross sectional survey, women who delivered 6 months ago	n= 2420 (slum dwellers)<br>Home deliveries= 22.62%	Not reported

For peer review only - http://bmjopen.bmj.com/site/about/guidelines.xhtml

Qualitative data from our study suggest that there was a lack of perceived risk among women and their family members, and priority was given to other domestic responsibilities over safe delivery. This may have been particularly relevant among multiparous women. A low literacy level of the HOH was another important predictor indicating the need to raise awareness of safe birthing practices. The role of CHWs in improving maternal and neonatal health indicators by increasing awareness is well known.[25] Though the Delhi State Health Mission has deployed Accredited Social Health Activists (ASHA) in the urban areas replicating the rural model, a recent evaluation has shown several implementation gaps.[26]

Women in our study were deterred from delivering in hospital out of fear and embarrassment. Traditionally, Indian women have delivered at homes surrounded by close family members. There is evidence to show that the support of a female relative during the birth process is beneficial and leads to better birth outcomes.[27] The state of Tamil Nadu has implemented a successful birth companion scheme through its public health system which addresses the issues of social support, fear and embarrassment.[28] This model provides a prototype that may be replicated, with suitable context-specific modifications, to address this important barrier to institutional deliveries.

Koblinsky et al[29] analysed national level data from several countries which reduced the MMR drastically since 1950 and showed that maternal deaths could be reduced by providing training to *dais or* professionals developing a partnership with *dais*, however it required effective outreach and referral mechanisms that support traditional system of birthing. Experiences from Malaysia and Sri Lanka show that, women are willing to move from home based to facility-

For peer review only - http://bmjopen.bmj.com/site/about/guidelines.xhtml

based delivery if transport and services are made free for all, improve awareness and also ensured quality of services at facilities.[29]

Current initiatives by the government aimed at improving MCH indicators of the urban poor do not directly address some of the key elements identified in our study. Identification and mapping of the most vulnerable populations within the city, sensitization of health professionals to the needs and fears of women, improving reach of UCHW to the marginalized, empowering women with information regarding their healthcare entitlements, provision of BPL, ration and RSBY cards to the neediest are some of the key issues that need focused and aggressive implementation.

It is important for health departments to; strengthen the supply side, be more accessible to those who need them the most and establish faith among the community. India needs to explore innovative ways at all levels of care to make delivery practices safer. There is hope that the urban health situation will improve in the coming years with the NUHM, if we intervene at the individual, community, system and policy level. The ANCHUL project and similar such endeavours all over the country will feed to provide innovative scalable strategies for the betterment of our urban community.

Acknowledgements: We would like to acknowledge all the families who volunteered to provide data for this survey. We thank the efforts of field staff (Field coordinators, Field supervisor, and Field interviewers), Data manager and Data entry operators.

Contributorship statement: SGJ and ND conceived and designed the study with additional inputs from AHS and SZ. ND analysed the quantitative data and prepared the first draft of the manuscript. SMG analysed the qualitative data and wrote the first draft of the qualitative

For peer review only - http://bmjopen.bmj.com/site/about/guidelines.xhtml

findings. GA and AS supervised data collection and commented on drafts of the manuscript. HN and AHS commented on drafts of the manuscript. SZ is the Principal Investigator of ANCHUL, had overall responsibility for ANCHUL, and commented on drafts of the manuscript. All authors contributed to critique and modification of the manuscript, read and approved the final version. ND had full access to all the data in the study and had final responsibility for the decision to submit for publication.

Funding: WHO**,** Geneva (Alliance for Health Policy and Systems Research (HSS/AHPSR), The Special Programme for Research and Training in Tropical Diseases (IER/TDR), The Special Programme of Research, Development and Research Training in Human Reproduction (HRP), Child and Adolescent Health and Development (FCH/CAH)) (Grant 2011/139172-0). The funders did not have any role in study design; the collection, analysis, and interpretation of data; the writing of the article or the decision to submit this article for publication

Ethics Approval: The study protocol was approved by Health Ministry Screening Committee of the Government of India, Institutional ethics committees of the Public Health Foundation of India, All India Institute of Medical Sciences, and Harvard School of Public Health.

Data sharing statement: All unpublished data related to this research project is available with the authors.

For peer review only - http://bmjopen.bmj.com/site/about/guidelines.xhtml

REFERENCES

1. WHO,UNICEF,UNFPA and The World Bank estimates: Trends in Maternal Mortality: 1990 to 2010, http://whqlibdoc.who.int/publications/2012/9789241503631_eng.pdf, (Accessed on 30th Decemebr, 2012).

2. World health statistics. http://apps.who.int/gho/data/node.main.214 (Accessed on December 2012).

3. Available from http://www.unescap.org/stat/apex/2/apex2_s.2_india_mdgs.pdf (accessed on 1st June, 2012).

4. National Population Policy – 2000. New Delhi: Ministry of Health and Family Welfare Government of India2000.

5. Women and health: Health service coverage data by country: http://apps.who.int/gho/data/node.main.223, (Accessed December 30th, 2012).

6. Registrar General of India. Sample registration system- Statistical Report 2010. New Delhi: Vital Statistics Division, Ministry of Home Affairs, Government of India; 2010.

7. Janani Suraksha Yojna. http://www.mohfw.nic.in/NRHM.htm (accessed on Jan 2013).

8. Lim SS, Dandona L, Hoisington JA, James SL, Hogan MC, Gakidou E. India's Janani Suraksha Yojana, a conditional cash transfer programme to increase births in health facilities: an impact evaluation. Lancet 2010;**375**(9730):2009-23 doi: 10.1016/S0140-6736(10)60744-1[published Online First: Epub Date]|.

9. Randive B, Diwan V, De Costa A. India's Conditional Cash Transfer Programme (the JSY) to Promote Institutional Birth: Is There an Association between Institutional Birth Proportion and Maternal Mortality? PLoS One 2013;**8**(6):e67452 doi: 10.1371/journal.pone.0067452[published Online First: Epub Date]|.

10. Hazarika I. Women's reproductive health in slum populations in India: evidence from NFHS-3. Journal of urban health : bulletin of the New York Academy of Medicine 2010;**87**(2):264-77 doi: 10.1007/s11524-009-9421-0[published Online First: Epub Date]|.

11. District Level Household and Facility Survey-Factsheet, Delhi, Available at http://www.rchiips.org/pdf/rch3/state/Delhi.pdf.

12. National Urban Health Mission, http://www.uhrc.in/module-ContentExpress-display-ceid-95.html (Accessed on Dec 31, 2012).

For peer review only - http://bmjopen.bmj.com/site/about/guidelines.xhtml

13. Available at http://pipnrhm-mohfw.nic.in/index_files/non_high_focus_small/Delhi/Executive_Summary_2011-12_final.pdf.

14. NCR policy zones, Demographic profile and Settlement pattern (2001) Available at http://ncrpb.nic.in/pdf_files/SG1.pdf.

15. JJ cluster list; Available from http://delhishelterboard.in/main/. As accessed on 11th July,2012.

16. Vyas S, Kumaranayake L. Constructing socio-economic status indices: how to use principal components analysis. Health policy and planning 2006;**21**(6):459-68 doi: 10.1093/heapol/czl029[published Online First: Epub Date]|.

17. Thind A, Mohani A, Banerjee K, Hagigi F. Where to deliver? Analysis of choice of delivery location from a national survey in India. BMC Public Health 2008;**8**:29 doi: 10.1186/1471-2458-8-29[published Online First: Epub Date]|.

18. Dasgupta S, Das P, Mandal NK, Karmakar PR, Ray RP, Mandal AK. A study on intranatal care practices in a district of West Bengal. Indian J Public Health 2006;**50**(1):15-8

19. Rahi M, Taneja DK, Misra A, Mathur NB, Badhan S. Newborn care practices in an urban slum of Delhi. Indian J Med Sci 2006;**60**(12):506-13

20. Agarwal P SM, Garg S. Maternal health care utilization among women in an urban slum in Delhi. Indian Journal of Community Medicine 2007;**32**(3):203-05

21. Agarwal S, Sethi V, Srivastava K, Jha PK, Baqui AH. Birth preparedness and complication readiness among slum women in Indore city, India. J Health Popul Nutr 2010;**28**(4):383-91

22. Das S, Bapat U, More NS, Chordhekar L, Joshi W, Osrin D. Prospective study of determinants and costs of home births in Mumbai slums. BMC Pregnancy Childbirth 2010;**10**:38 doi: 10.1186/1471-2393-10-38[published Online First: Epub Date]|.

23. Khan Z, Mehnaz S, Khalique N, Ansari MA, AR S. Poor perinatal care practices in urban slums: possible role of social mobilization networks Indian J Community Med 2009;**34**(2):102-07

24. Singh PK, Rai RK, Singh L. Examining the effect of household wealth and migration status on safe delivery care in urban India, 1992-2006. PLoS One 2012;**7**(9):e44901 doi: 10.1371/journal.pone.0044901[published Online First: Epub Date]|.

25. Lassi ZS, Haider BA, Bhutta ZA. Community-based intervention packages for reducing maternal and neonatal morbidity and mortality and improving neonatal outcomes. Cochrane

For peer review only

For peer review only - http://bmjopen.bmj.com/site/about/guidelines.xhtml

Database Syst Rev 2010(11):CD007754 doi: 10.1002/14651858.CD007754.pub2[published Online First: Epub Date]|.

26. Sixth Common Review Mission Report. Available at http://nhsrcindia.org/pdf_files/resources_thematic/Health_Sector_Overview/NHSRC_Contribution/Final_6thCRM_Book_NRHM.pdf. Accessed on May 2013.

27. Hodnett ED, Gates S, Hofmeyr GJ, Sakala C. Continuous support for women during childbirth. Cochrane Database Syst Rev 2012;**10**:CD003766 doi: 10.1002/14651858.CD003766.pub4[published Online First: Epub Date]|.

28. Padmanaban P, Raman PS, Mavalankar DV. Innovations and challenges in reducing maternal mortality in Tamil Nadu, India. J Health Popul Nutr 2009;**27**(2):202-19

29. Koblinsky M, Kureshy N. Safe motherhood case studies: learning with stakeholders in South Asia--an introduction. J Health Popul Nutr 2009;**27**(2):89-92

For peer review only

For peer review only - http://bmjopen.bmj.com/site/about/guidelines.xhtml

STROBE 2007 (v4) Statement—Checklist of items that should be included in reports of *cross-sectional studies*

Section/Topic	Item #	Recommendation	Reported on page #
**Title and abstract**	1	(*a*) Indicate the study's design with a commonly used term in the title or the abstract	2
		(*b*) Provide in the abstract an informative and balanced summary of what was done and what was found	2
**Introduction**			
Background/rationale	2	Explain the scientific background and rationale for the investigation being reported	5
Objectives	3	State specific objectives, including any prespecified hypotheses	6
**Methods**			
Study design	4	Present key elements of study design early in the paper	7
Setting	5	Describe the setting, locations, and relevant dates, including periods of recruitment, exposure, follow-up, and data collection	7
Participants	6	(*a*) Give the eligibility criteria, and the sources and methods of selection of participants	8
Variables	7	Clearly define all outcomes, exposures, predictors, potential confounders, and effect modifiers. Give diagnostic criteria, if applicable	8
Data sources/ measurement	8*	For each variable of interest, give sources of data and details of methods of assessment (measurement). Describe comparability of assessment methods if there is more than one group	NA
Bias	9	Describe any efforts to address potential sources of bias	*8*
Study size	10	Explain how the study size was arrived at	9
Quantitative variables	11	Explain how quantitative variables were handled in the analyses. If applicable, describe which groupings were chosen and why	10
Statistical methods	12	(*a*) Describe all statistical methods, including those used to control for confounding	10
		(*b*) Describe any methods used to examine subgroups and interactions	NA
		(*c*) Explain how missing data were addressed	NA
		(*d*) If applicable, describe analytical methods taking account of sampling strategy	10
		(*e*) Describe any sensitivity analyses	NA
**Results**			
Participants	13*	(a) Report numbers of individuals at each stage of study—eg numbers potentially eligible, examined for eligibility, confirmed eligible, included in the study, completing follow-up, and analysed	11
		(b) Give reasons for non-participation at each stage	13
		(c) Consider use of a flow diagram	13
Descriptive data	14*	(a) Give characteristics of study participants (eg demographic, clinical, social) and information on exposures and potential confounders	14
		(b) Indicate number of participants with missing data for each variable of interest	14
Outcome data	15*	Report numbers of outcome events or summary measures	16

For peer review only

Main results	16	(*a*) Give unadjusted estimates and, if applicable, confounder-adjusted estimates and their precision (eg, 95% confidence interval). Make clear which confounders were adjusted for and why they were included	16
		(*b*) Report category boundaries when continuous variables were categorized	16
		(*c*) If relevant, consider translating estimates of relative risk into absolute risk for a meaningful time period	NA
Other analyses	17	Report other analyses done—eg analyses of subgroups and interactions, and sensitivity analyses	NA
**Discussion**			
Key results	18	Summarise key results with reference to study objectives	20
Limitations	19	Discuss limitations of the study, taking into account sources of potential bias or imprecision. Discuss both direction and magnitude of any potential bias	21
Interpretation	20	Give a cautious overall interpretation of results considering objectives, limitations, multiplicity of analyses, results from similar studies, and other relevant evidence	21
Generalisability	21	Discuss the generalisability (external validity) of the study results	22
**Other information**			
Funding	22	Give the source of funding and the role of the funders for the present study and, if applicable, for the original study on which the present article is based	24

*Give information separately for cases and controls in case-control studies and, if applicable, for exposed and unexposed groups in cohort and cross-sectional studies.

Note: An Explanation and Elaboration article discusses each checklist item and gives methodological background and published examples of transparent reporting. The STROBE checklist is best used in conjunction with this article (freely available on the Web sites of PLoS Medicine at http://www.plosmedicine.org/, Annals of Internal Medicine at http://www.annals.org/, and Epidemiology at http://www.epidem.com/). Information on the STROBE Initiative is available at www.strobe-statement.org.

For peer review only

Why women choose to give birth at home: A situational analysis from urban slums of Delhi

Journal:	*BMJ Open*
Manuscript ID:	bmjopen-2013-004401.R1
Article Type:	Research
Date Submitted by the Author:	01-May-2014
Complete List of Authors:	Devasenapathy, Niveditha; Indian Institute of Public Health-Delhi, Public Health Foundation of India,<br>George, Mathew; Public Health Foundation of India, Indian Institute of Public Health-Delhi<br>Ghosh-Jerath, Suparna; Public Health Foundation of India, Indian Institute of Public Health-Delhi<br>Singh, Archna; All India Institute of Medical Sciences, Department of Biochemistry<br>Negandhi, Himanshu; Public Health Foundation of India, Indian Institute of Public Health-Delhi<br>Alagh, Gursimran; Public Health Foundation of India, Indian Institute of Public Health-Delhi<br>Shankar, Anuraj; Harvard School of Public Health, Department of Nutrition<br>Zodpey, Sanjay; Public Health Foundation of India, Indian Institute of Public Health-Delhi
**Primary Subject Heading**:	Public health
Secondary Subject Heading:	Health services research, Qualitative research
Keywords:	PUBLIC HEALTH, QUALITATIVE RESEARCH, Quality in health care

SCHOLARONE™
Manuscripts

For peer review only - http://bmjopen.bmj.com/site/about/guidelines.xhtml

Why women choose to give birth at home: A situational analysis from urban slums of Delhi

Niveditha Devasenapathy[1], MBBS, M.Sc, Mathew Sunil George[1], MPH, Suparna Ghosh Jerath[1], PhD, Archna Singh[2], PhD, Himanshu Negandhi[1], MD, Gursimran Alagh B.Sc[1], Anuraj H Shankar[3], D.Sc, Sanjay Zodpey[1] MD, PhD

[1] Indian Institute of Public Health, Delhi, Public Health Foundation of India, Gurgaon, India,
[2]Department of Biochemistry, All India Institute of Medical Sciences, New Delhi, India
[3]Department of Nutrition, Harvard School of Public Health, Boston, Massachusetts, USA

Address for correspondence

Dr Niveditha Devasenapathy MBBS, MSc
Associate Professor
Indian Institute of Public Health- Delhi
Plot No. 34, Sector 44, Institutional Area, Gurgaon 122002, India
Phone: 91-124-4722922, Fax : 91-124-4722901
email:niveditha@iiphd.org

Key Words: Reproductive Health, Access to Health Care, Health Promotion, Needs Assessment, Maternal Health

Word count
Abstract: 273
Main text: 4128

For peer review only

For peer review only - http://bmjopen.bmj.com/site/about/guidelines.xhtml

ABSTRACT

Objectives: Increasing institutional births is an important strategy for attaining MDG-5. However, rapid growth of low-income and migrant populations in urban settings in low and middle income countries, including India, presents unique challenges for programs to improve utilization of institutional care. Better understanding of the factors influencing home or institutional birth among the urban poor is urgently needed to enhance program impact. To measure prevalence of home and institutional births in an urban slum population and identify factors influencing these events.

Design: Cross-sectional survey using quantitative and qualitative methods

Setting: Urban poor settlements in Delhi, India

Participants: A house-to-house survey was conducted of all households in three slum-clusters in north-east Delhi (n=32,034 individuals). Data on birthing place and socio-demographic characteristics were collected using structured questionnaires (n=6092 households). Detailed information on pregnancy and postnatal care was obtained from women who gave birth in the last 3 months (n=160). Focus group discussions and in-depth interviews were conducted with stakeholders from the community and health-care facilities.

Results: Of 824 women who gave birth in the previous year, 53% [95%CI 49.7-56.6] had given birth at home. In adjusted analyses, multiparity, low literacy and migrant status were independently predictive of home births. Fear of hospitals (36%), comfort of home (20.7%) and lack of social support for child-care (12.2%) emerged as the primary reasons for home births.

For peer review only - http://bmjopen.bmj.com/site/about/guidelines.xhtml

Conclusions: Home births are frequent among the urban poor. This study highlights the urgent need for improvements in the quality and hospitality of client services, needs for family support as key modifiable factors affecting over two thirds of this population. These findings should inform the design of strategies to promote institutional births.

Article Summary

Strengths and Limitations of the study

- This survey covered a large number of households (n= 6092) households living in 3 urban poor settlements of Delhi
- Both qualitative and quantitative methods were used to capture reasons for home births.
- Though the slum cluster was not a random sample from all the slum clusters in Delhi they were representative of the urban poor settlements.
- Concurrent health facility assessment was not done which would have helped to understand additional supply-side factors.

For peer review only

INTRODUCTION

Increasing institutional births is a key global strategy to reduce maternal and new-born mortality. Many countries, including India, have established incentive programs and policies to enhance institutional births. However, the rapid growth of low-income urban populations presents unique challenges to these programs such as lower knowledge of local services and registration processes, lack of support from an extended family and transient residence. With around 40% of urban population in low and middle income countries residing in low-income urban settlements, more focused efforts are required to improve institutional birth rates in these settings.

Most maternal deaths are centred around the intra-partum and immediate postpartum period [1 2] and for countries with high burden of maternal mortality and morbidity, facility based birthing is found to be efficient and sustainable compared to scaling up community based safe birthing programs.[3 4] An evaluation study of the safe motherhood program in Indonesia showed that irrespective of level of socioeconomic status and place of residence (urban vs. rural) increasing the number of deliveries by Skilled Birth Attendant (SBA) did not reduce maternal mortality if most births took place at home. [5] In contrast, programs that enhanced facility-based births in Malaysia and Sri Lanka resulted in marked reduction in maternal and neonatal deaths.[6] With the overwhelming evidence for scaling up quality health centre based intra-partum care in improving maternal and neonatal survival, it is important for each country and local administration to understand barriers at community and facility level that affect the access to facilities and provision of quality services.

For peer review only - http://bmjopen.bmj.com/site/about/guidelines.xhtml

India currently accounts for about a fifth of all maternal and new-born deaths worldwide [7], and approximately one third of the population currently lives in urban areas, and growing to nearly one half by 2030. The MDG report has flagged the slow progress of India in reducing child mortality and improving maternal health [8], with the latest WHO statistics showing that only half of expectant mothers in India complete four antenatal care (ANC) visits and give birth in the presence of a SBA. [9] Overcoming barriers to institutional births among the urban poor is therefore crucial.

The National Rural Health Mission (NRHM) of India therefore launched the Janani Suraksha Yojana (JSY) program in 2005 with the goal of reducing maternal and neonatal mortality by promoting institutional births among poor pregnant women.[10] An evaluation of this conditional cash transfer scheme in 2007-08 showed an increase in ANC visits and institutional births.[11] However, this has not translated to reduction in MMR possibly due to unaddressed issues of non-financial access barriers and sub-optimal ANC, delivery and postnatal care.[12] In addition, unique issues faced by urban poor were not specifically addressed by the JSY program. As mentioned above, currently 30% of India population are living in cities. Delhi is one of the most densely populated cities in the world, and attracts nearly 500,000 migrants every year with most settling in urban poor habitations.

According to the National Family Health Survey (NFHS 3) survey conducted in 2005-06, only 44 % of births were institutional among the urban poor of Delhi as compared to the urban average of 67.5%.[13] The District Level Household and Facility Survey (2007-08) survey showed that overall, 71% of pregnant women had at least 3 ANC visits. While 68% of births were

For peer review only - http://bmjopen.bmj.com/site/about/guidelines.xhtml

institutional in the city as a whole, only 38% institutional deliveries were reported in slum areas.[14] A governmental initiative aimed at correcting this inequity is the National Urban Health Mission (NUHM) which makes primary health care services available to the urban poor.[15],[16] The success of this mission will depend on identifying and targeting interventions directed towards the most vulnerable. One of the aims of this study is to determine the prevalence of home and institutional births among women living in urban poor settlements in Delhi and identify reasons influencing their choice.

METHODS

This study is part of the formative phase and situational analysis for the ANCHUL (Ante Natal and Child Health care in Urban Slums, CTRI/2011/091/000095) trial, an implementation research project aimed to develop, implement and evaluate the impact of an intervention package delivered through an urban community healthcare worker (UCHW). ANCHUL aims to increase access to health care facilities for birthing and improve maternal, neonatal, child healthcare (MNCH) practices in urban slums of Delhi. This study aimed to conduct an in-depth situational analysis on utilization and quality of MNCH care using quantitative and qualitative methods. The information obtained will guide the development of the community based intervention package to be delivered by the UCHW as part of the ANCHUL trial.

Study Setting

Of the 16.7 million people living in Delhi, 52% reside in poor habitations.[17] The north-east district of Delhi contributes 11%, about one fifth, to this population with 44 registered slums.[18] This district has the highest home birth rate.[14] We conducted a rapid survey in 17 slum clusters to obtain information on number of households, water supply, sanitation,

For peer review only - http://bmjopen.bmj.com/site/about/guidelines.xhtml

presence of schools, healthcare facility, and distance from nearest government hospital. The clusters were then stratified into two categories of vulnerability based on the above characteristics. We then randomly selected three vulnerable slum clusters namely Buland Masjid (BM), CPJ and Chanderpuri (CP*)*. These slums had tarred roads within and had access to maternal child health care dispensaries within a distance of five kms. The study protocol was approved by Health Ministry Screening Committee of the Government of India, institutional ethics committees of the Public Health Foundation of India, All India Institute of Medical Sciences, WHO Geneva, and Harvard School of Public Health.

Data collection

Quantitative survey

After lane mapping the clusters, all households were included in the survey. We identified pregnant women (in their 2nd and 3rd trimesters), recently delivered women (RDW, i.e., those who had delivered in the last 3 months) and households with under-5 children. The purpose of the survey was explained to a household member above 18 years of age and all questionnaires were administered after obtaining informed consent by trained field interviewers. All survey tools were in local language and were piloted and modified for content and clarity. Information on family details, socio-demographic status, place of childbirth (in women who had given birth in past 1 year), and information on any maternal and child deaths within households in past 1 year, was obtained using paper forms. All refusals and non-responses were documented. We re-visited the households of 160 RDW and collected detailed information about ANC, delivery, immediate post-natal period, new born care practices and diet of the mother. Data were checked for completion before entering into a structured database management system

For peer review only - http://bmjopen.bmj.com/site/about/guidelines.xhtml

(Microsoft Access 2010) with inbuilt range and internal consistency checks. Information from RDW was edited and validated by double data entry.

Qualitative data

The categories of respondents in *table 1* were identified as relevant for data collection in this study. Households were informed that focus group discussions (FGDs) would be held in the community and a general invitation was given. Permissions were sought from local community and religious leaders. Local public and private health care providers were approached and permissions were sought for in-depth interviews (IDI). The FGD and IDI guides were piloted to refine the topic guides to enable them to generate data that was relevant to the study objectives. The main topics that were explored in the FGDs and interviews are shown in *table 1*. The venue for data collection was agreed upon based on the respondent's convenience. One interviewer facilitated the discussions while a second took notes. Based on responses from the community, health care facilities and traditional birth attendants who served the locality were identified and approached. Written informed consent was obtained from all participants before the FGDs and IDIs, which were digitally voice-recorded.

Sample size justification

For estimating the number of households to be interviewed, institutional birth was considered the key outcome variable. Assuming prevalence of institutional births as 33% in urban slums of Delhi[16], we needed to interview 780 women who gave birth in the past year to obtain prevalence estimates with 10% relative precision. Assuming a crude birth rate of 25/1000 (national average is 21/1000), a population of 30,000 was to be covered to identify at least 750 childbirths in the past year.

For peer review only - http://bmjopen.bmj.com/site/about/guidelines.xhtml

Table 1: Themes covered for qualitative data

Category of participants	Method of data collection	Key themes covered
**Community**<br>• Pregnant women (n= 5)<br>• Recently delivered women (n=6)<br>• Mother of under 5 children (n=6)<br>• Mother in laws (n=5)<br>• Husbands (n=4)	Focus Group Discussions(FGDs)<br><br>Venue: Schools, NGO, Madrassa(religious place) and anganwadi centres*	• Health and nutritional status<br>• Cultural practices for nutrition during pregnancy<br>• Care seeking behaviour during pregnancy<br>• Barriers to accessing care during pregnancy<br>• Quality of care experienced in various health care settings (public and private)
**Health Care Providers**<br>• Public Health system (n=6)<br>• Private (n=5)<br>• Others (n=4) (AWW, TBAs)	In depth interviews<br>Venue: Clinics of health care providers or homes of key informants	• Care seeking pattern among the community during pregnancy<br>• Challenges to improving maternal and child health among urban poor<br>• Feasibility of proposed intervention
**ANC Clinic attendees** (n=9)	Exit interviews (pre and post ANC check-ups)<br>Venue: Clinics	• Experience of care during ANC visit<br>• Satisfaction levels of the individual about care

AWW: Anganwadi workers, TBA: Traditional birth attendants *(Dai)*
*The word Anganwadi means "courtyard shelter" in Hindi. They were started by the Indian government in 1975 as part of the Integrated Child Development Services program to combat child hunger and malnutrition.

Data analysis

Quantitative data

Data were analysed using Stata 11 (Stata Corporation, College Station, TX). Descriptive statistics were used to provide a cluster, household and individual level profiles of the study population. Household survey data were analysed accounting for clustering at the slum level to control for both inter and intra-cluster variance. We used principal component analysis to compute household Socioeconomic Scales (SES). Dwelling characteristics, household income and household assets were included in this composite scale.[19] We used multivariate random

For peer review only - http://bmjopen.bmj.com/site/about/guidelines.xhtml

effects logistic regression to estimate the association of demographic variables with home births. Crude and adjusted odds ratios were calculated with 95% confidence intervals. For data from RDWs, Pearson chi-squared was used for categorical variables and t-tests for comparison of continuous variables.

Qualitative data

Verbatim transcripts were prepared in a standardized format that included basic demographic information of the participants and the interviewer's own observations within one week of conduct of IDI/FGD. Transcripts were uploaded to a software Atlas ti 6.1 (Scientific Software Development, City West, Berlin) and coded line-by-line using detailed themes and sub-themes that emerged from the data. After an initial round of coding with a representative sample of transcripts, the list of codes that were generated was reviewed in order to develop a structured code list which was then applied to the remaining transcripts. Illustrative quotations that captured the key issues reported by the participants have been included in the results.

RESULTS

Of the 6348 households in the three defined clusters, 6092 (96%) were interviewed between December 2011 and March 2012, covering a total population of 32,034. Nine households refused to participate and 247 did not respond (locked houses) (figure 1). A total of 25 FGDs and 13 in-depth interviews were conducted in January and February 2012. The number of respondents in each FGD ranged from 7-12 members.

For peer review only - http://bmjopen.bmj.com/site/about/guidelines.xhtml

Population and cluster characteristics

The adult male to female ratio was 1000:825. Fifty eight percent of the population were migrants from Uttar Pradesh (73%) and Bihar (16%). Eighty percent were living in the same locality for > 5 years. Of the total population, women of reproductive age (15-49 years) accounted for 25%, and 16.6% were under-5 children. The area was served by one referral hospital situated within a distance of 5kms, two outpatient dispensaries, 17 private clinics (registered and unregistered with the Medical Council) and one laboratory within the clusters. The areas also have access to two referral hospitals situated at distance of about 10 kms.

Household characteristics

The median family size was 5 (IQR 4, 7) predominantly living as nuclear families (79.4%) and 63% of houses were self-owned. The head of the household (HOH) was the one considered as the decision-maker but was not necessarily the primary wage earner. Fifty nine percent of HOHs were illiterate and were unskilled labourers. Ration cards, Below Poverty Line cards (BPL) and Rashtriya Swasthya Bima Yojna (RSBY) cards that are needed for claiming government run health schemes were possessed by only 50%, 31% and 24% of households respectively. The majority of the households (82%) lived in single-roomed concrete houses with cemented or tiled flooring. Most houses (95%) had access to toilets within the household or community. A detailed socio-demographic profile of the study population is presented in *Table 2*. The household characteristics of the RDW and the overall population in the study area were similar indicating that our subsample households were representative of this area. Fifteen maternal deaths, 21 still-births and 41 under-5 child deaths were reported for the previous year. Of the total child deaths, 22 were in the neonatal period.

For peer review only - http://bmjopen.bmj.com/site/about/guidelines.xhtml

Table 2: Characteristics of households in the study area and households of women who gave birth in previous 3 months

Demographic characteristics	House to House survey (n=6092 HH from 3 clusters	Mothers who recently gave birth (n=160)
Median HH size (IQR)	5(4,7)	5 (4,7)
Family type (%)		
Nuclear	4834(79.4)	115 (71.9)
Joint	944(15.5)	43 (26.9)
Extended	313 (5.1)	2 (1.3)
Spoken language (%)		
Hindi	5328 (87.5)	145 (90.6)
Urdu	624(10.2)	15 (9.4)
Others	140(3.2)	-
Religion (%)		
Hindu	1822(29.9)	42 (26.3)
Muslim	2475 (69.6)	118 (73.8)
Others	33(0.5)	-
Caste category (%)		
General	2546 (41.8)	77 (48.1)
Other Backward Caste	2553 (41.9)	58 (36.3)
Scheduled caste/Scheduled Tribe	932 (15.3)	22 (13.8)
Do not want to answer	4 (0.1)	-
Do not know	57 (0.9)	3 (1.9)
Illiterate women in reproductive age group (%)	(n= 8056) 4122(51.2)	85 (53.1)
Literacy level of HOH (%)		
Illiterate	3561(58.5)	93 (58.1)
Literate but no formal education	196(3.2)	4 (2.5)
Schooling	2129(36.4)	62 (38.8)
College	115(1.89)	1 (0.6)
Occupation of HOH (%)		
Unskilled	2805 (46.3)	77 (48.4)
Skilled	1378 (22.7)	36 (22.6)
Office work	867 (14.3)	19 (12)
Professional	55 (0.91)	-
Not working	955 (15.76)	27 (17)
Median HH income in INR (IQR)	4000(3000-6500)	4000 (3000-7000)
Median HH income in USD (IQR)	76.2(57.1- 123.8)	76.2 (57.1-133.5)
Own house (%)	3829 (62.9)	101 (63.1)
Ration card (%)		
Don't have	2994 (49.2)	91 (56.9)
White	1173 (19.3)	22 (13.8)
Yellow	1196 (19.6)	28 (17.5)
Pink	686 (11.3)	18 (11.3)

For peer review only - http://bmjopen.bmj.com/site/about/guidelines.xhtml

Do not want to answer	43 (0.7)	1 (0.6)
BPL card (%)	1903 (31.2)	47 (29.4)
RSBY card (%)	1461 (24)	36 (22.5)
% of HH who are staying in the current locality in years		
< 1	660 (10.8)	13 (8.1)
1-2	238 (3.9)	11 (6.9)
3-5	330 (5.4)	12 (7.5)
>5	4864 (79.8)	124 (77.5)
% belonging to Delhi	3572(57.49)	93 (58.1)
% of HH who migrated but living in Delhi		
< 1 year	261 (7.5)	2 (2.2)
1-2	123 (3.5)	3 (3.2)
3-5 years	195 (5.6)	2 (2.2)
>5 years	2920 (83.5)	86 (92.5)
Socioeconomic categories* (%)		
Lowest	1.976(32.45)	53 (33.1)
Middle	2,077 (34.11)	53 (33.1)
Highest	2,036 (33.44)	54 (33.8)
Distance of HH from nearest Maternal Child Health care centre		
<5 km	4882 (80.1)	125 (78.1)
5-10 km	1550 (18.9)	29 (18.1)
>10 km	60 (1)	6 (3.8)

HOH: Head of Houshold, BPL: Below Poverty Line card, RSBY: Rashtriya Swasthya Bhima Yojna(Health insurance scheme)*The scale is a composite of house type, floor, house ownership, separate kitchen, TV, Refrigerator, Mobile phone, washing machine, total HH income, Number of rooms by principal component analysis

Place of childbirth

Of the 824 women who gave birth in the previous year, 438 were home births [53.1%, 95%CI (49.1 - 56.6)] and of the remaining 386, 340 (88%) chose to give birth at a public hospital. Among the women who gave birth in the previous 3 months (n=160), a similar proportion [53.1 %, 95% CI (45.0- 61.0)] gave birth at home. Only 16.2% (12) of these mothers' availed cash incentive through JSY scheme *(table 3)*. Thirty six (48%) went to hospital due to initiation of labour pains, 32% due to development of complications, and 6.7% reported the reason that they had crossed expected date of delivery. The individual who was most influential in the decision for delivering at a hospital was most often (48%) the women herself, followed by the

For peer review only - http://bmjopen.bmj.com/site/about/guidelines.xhtml

husband (18.7%) or mother-in-law (17.3%). Irrespective of the place of delivery only 15% of these households were visited by a health worker within 48hrs of delivery and only 30% of women visited a health care facility after giving birth. Among those who gave birth in a facility 92% were satisfied with the services provided at the hospital. Women who gave birth at home were more likely to be multiparous, less likely to avail ANC in a public hospital and visit a facility during the post-partum period *(table 3).*

Predictors of home births

Among the 824 women who gave birth in the previous year, the following demographic characteristics were significantly associated with home births: Living in a rented house, low SES, low literacy of HOH, HOH being an unskilled labourer, migrants and multi-parity. Multi-parity [OR 1.96, 95% CI (1.44, 2.69)], literacy status of HOH [OR 0.71, 95%CI (0.53 0.97)] and migrant status [1.46, 95% CI (1.08, 1.97)] remained strong independent predictors of home births in multivariate analysis *(Table 4)*.

Table 3: Information on ANC and births obtained from recently delivered women

Characteristics*	Home births (n=85)	Institutional births (n=75)	p value
Mean age	24.7(4.31)	24.9(4.43)	0.79
>18yrs of age at marriage	72 (84.7)	64(85.3)	0.912
Mean family size	5.5(2.4)	5.5(2.3)	0.937
First child	15 (17.7)	26 (34.7)	**0.014**
Some ANC care	63 (74.1)	72 (96)	**<0.0001**
ANC at public hospital	41 (65)	61(84.7)	**< 0.0001**
ANC in first trimester	21 (33.33)	31 (43.1)	0.498
Some health problem during pregnancy	11 (17.5)	13 (18)	0.92
Satisfaction with ANC	46 (74.6)	65 (90.3)	0.046
Planned place of birth	73 (86)	66 (90.4)	0.38
Birth conducted by Doctor/ Nurse	15 (17.7)	74 (98.7)	**<0.0001**
Home visitation by	14 (16.5)	11 (14.7)	0.75

For peer review only - http://bmjopen.bmj.com/site/about/guidelines.xhtml

community health worker			
Post-partum visit to hospital	3 (3.53)	16 (21.33)	**0.001**

*All continuous variables are expressed as mean and one standard deviation; all proportions are expressed as percentages

Table 4: Predictors of home births

Characteristics (n=824)<br>438: Home births<br>386: Institutional delivery	p value (ignoring clustering)	Crude OR (95% CI), p value (accounted for clustering)	Adjusted* OR (95% CI), p value (LR test)
Buland Masjid<br>CPJ<br>Chaderpuri	<0.001	1 **<0.001**<br>0.42(0.30, 0.59)<br>0.69 (0.49, 96)	
Birth order second and above	<0.001	2.12 (1.57, 2.87) **<0.001**	1.96 (1.44, 2.69) **<0.001**
Lower SES<br>Middle SES<br>Highest SES	0.033	1<br>0.90 (0.64, 1.26) **0.011**<br>0.70 (0.50 0.98)	0.96 (0.66, 1.406) 0.68<br>0.91 (0.60, 1.38)
Joint families	0.057	0.78 (0.57, 1.05) 0.102	
Non-Muslims	0.004	0.77 (0.54, 1.1) 0.15	0.78 (0.54, 1.14) 0.21
Schooling of HOH	0.007	0.74 (0.55, 0.99) **0.04**	0.71 (0.53 0.97) **0.031**
Not working<br>Elementary job<br>Skilled job	0.024	1 **0.063**<br>1.63(1.08, 2.45)<br>1.42 (0.92, 2.17)	1 0.2<br>1.54 (1, 2.38)<br>1.40 (0.89, 2.21)
Own the house	0.004	0.76 (0.56, 1.02) **0.07**	0.94 (0.65, 1.34**)** **0.72**
Ration card possession	0.037	0.80 (0.60, 1.07) 0.14	
Not belonging to Delhi	<0.001	1.61(1.21, 2.15) **0.001**	1.46 (1.08, 1.97) **0.013**

*Adjusted for house ownership, SES, literacy of HOH, Occupation of HOH, belonging to Delhi and birth order

Reasons for choosing home birth

The majority of home births were pre-planned and 75% of these women had availed some ANC at a facility. Eighty two percent of the home births were conducted by a traditional birth attendant (*Dai*). The results from the quantitative and qualitative data, showed a high level of concordance for the reasons for choosing home births. Four major themes emerged as barriers to institutional births. Illustrative quotations from the transcripts are presented in *table 5*.

Table 5: Illustrative quotations for reasons for home birth

Fear and Embarrassment

For peer review only

For peer review only - http://bmjopen.bmj.com/site/about/guidelines.xhtml

"They prefer home deliveries as in many cases the doctor does not behave well with them. As soon as they enter, they are separated from their families. The doctor does not communicate to the relative if there is any complication. They stay for 1-2 days in the labour room; the relatives are outside they do not know what is happening. It is very scary for them. Also because of all these reasons, they will come only if it is life threatening. Even then they might prefer to go to someone who is local, who is more patient friendly, private practitioner who are non-judgemental who behave properly. They give more one to one care. They might not be qualified but it is more natural and human." ***Senior gynaecologist, Public health facility***<br><br>*"I'd prefer to have the baby at home. If you're in hospital, they don't even attempt to try for a natural birth. What's the use of having an operation if you can have a child the normal way? We get the check-ups done there, but we end up having the delivery at home".* ***Pregnant woman***<br><br>*"If you tell them to put you in a closed room to get a check - up, they tell you to just lie down and get it done right there. It's humiliating; you can't help but feel embarrassed. And if you don't feel embarrassed, other people around you will. They tell you that if you feel ashamed, go to a private hospital. It's a matter of dignity. There are men walking around as well, if a man catches a glimpse, it can create trouble at home".-****Pregnant woman***
Prior experience with hospitals<br><br>*"I had gone recently with my sister to a hospital……when I went there to deliver my baby, they just kept telling me 'keep pushing, keep pushing…" I got so scared I just left. I've had three children at home; I can manage a fourth the same way".* ***Recently delivered woman***
**Other children**<br><br>*"I have small children. If I have to go to the hospital, I have to lock the house and take the children along. Then if she delivers in the hospital, she may be admitted for at least 2 or 3 days depending on the situation. Even if it is a normal delivery it is at least a 2 day stay. How do I manage in such situations? To avoid all this one hopes that if all is well it is better to deliver at home itself. We can all be at home and kids need not have to go anywhere. I can also go for work."* ***Husband of pregnant woman***
**Opportunity costs**<br><br>*"Most of them earn daily wages, so they do not want to come to the hospital. They feel one day will go so they will lose their pay also...So the delivery can be at home if baby is okay. Male member does not want to involve himself in all these things, either if there is an elder woman in the house or neighbourhood who conducts the delivery...Even at the hospital they do not want to stay they say that they have to go to work, or how will they earn for tomorrow's food because they are working on daily wages.."* ***Senior gynaecologist, public health facility***

Fear and embarrassment

For peer review only - http://bmjopen.bmj.com/site/about/guidelines.xhtml

Fear and embarrassment associated with giving birth in hospitals were reported as the most important reasons for giving birth at home during FGDs and IDIs and was also reported as the key reason (35%) among the 85 RDW surveyed. Fear was due to being alone in unfamiliar surroundings and fear of surgical intervention. In addition to fear, women felt it was embarrassing and uncomfortable for them to be in the presence of 'strangers' during a very vulnerable time. The lack of privacy coupled with the absence of any family member by their side was in stark contrast to the 'safe and reassuring environment' at their homes during the birthing process.

Prior experience with hospitals

Prior experience of self, friends, neighbours or a family member played an important role in choosing home or hospital birth. Positive experiences reinforced the message that hospitals were a safe and welcoming place as opposed to negative experiences (such as, perceived improper care and rude behaviour of hospital staff). The health care providers interviewed indicated that high patient load at hospitals lead to lack of individual attention and inadequate care.

Domestic responsibilities

Being in an unfamiliar neighbourhood, the absence of extended family to help with childcare and traditional lack of involvement of men in childcare made women reluctant to leave children at home and get admitted to hospital. In the survey, 10% of those who had home births cited lack of help with childcare as a reason.

Opportunity costs

For peer review only - http://bmjopen.bmj.com/site/about/guidelines.xhtml

Though most services at the hospital were provided free of cost, opportunity costs in the form of lost wages for the earning member, cost of food for the family and travel, dissuaded some women from delivering in hospital. Although only 6% mentioned this as a reason in our survey, this emerged as a factor in the qualitative analysis. However, direct costs were not one of the reasons cited for not opting for hospital births.

DISCUSSION

Our study showed a high prevalence of home births conducted by *TBA* among the urban poor of north-east district of Delhi. Fear of surgical procedures, unfamiliarity with hospital surroundings, lack of help for childcare and loss of wages, were some of the reasons that drove women to choose home briths. Other predictors of home births were low literacy, higher parity and migrant status. Concordance between results derived from qualitative and quantitative data lends greater credibility to these findings. *Figure 2* presents a conceptual framework based on the study findings which could help us to design strategies for some of the modifiable factors.

The prevalence and reasons for home births in our study was similar to that found in most other urban surveys [13 14 20-26] from India *(Table 6).* In the Mumbai slum study[25], the prevalence varied from 6-16% across 48 slum clusters. Tradition was the most important reason behind home births in this study. Apart from the predictors that we identified, poor housing, lack of water supply and hazardous location were associated with home births in the Mumbai study, indicating that apart from individual and household level factors, the type of

For peer review only - http://bmjopen.bmj.com/site/about/guidelines.xhtml

neighbourhoods also played a role. One of the limitations of our study is that we could not evaluate cluster level predictors of home births as we included only three clusters in this study.

Migrant status was one of the important determinants of home births in our study. In an analyses using NFHS data, Singh et al[27] reported that urban poor migrants were at highest risk of unsafe birthing practices in contrast to non-poor, non-migrants who were at least risk. In our study sample almost 60% of the households were migrants from neighbouring states, but most living in Delhi for more than 5 years. In spite of this, these households were less likely to possess ration, BPL or RSBY cards which are required for availing entitlements to healthcare.

Quality of care did not figure in the discussions as a factor for choosing home births. The quantitative data also confirm that majority of women who visited hospital for ANC and for birthing were quite satisfied with the services offered. We hypothesize that since the focus on facility based birthing and offering free services to enable the same is comparatively new to the urban poor community, the community has not reached the stage of assessing the quality of care and using that as a factor in making a decision on where to deliver. Based on our observation of the facilities there is lot of scope for improvement in the services being offered suggesting a gap between what level of services women perceive they are entitled to, and what they actually receive.

The discomfort of hospitals expressed by those who gave birth at home could be attributed to the overburdening of referral hospitals leading to lack of personalized care. Antenatal care is provided at dispensaries, maternal and child care centres (MCH), secondary level and referral hospitals. However, MCH centres cater to deliveries of multigravida only, and all primigravida are referred to the secondary level or referral hospitals leading to increased patient load at

For peer review only - http://bmjopen.bmj.com/site/about/guidelines.xhtml

these centres. Initiatives to decentralize care to reduce burden on referral hospitals by upgrading the MCH centres has been rather slow. It might be possible that other supply-side issues could have also contributed to this level of dissatisfaction, but due to delay in obtaining permissions, we were unable to conduct facility assessment to identify the potential causes.

Table 6: Prevalence and reasons for home births from urban surveys in India since 2000

Author, year of publication (ref)	Study area and target population/ Study design	Sample size and prevalence of home births	Reasons for home home births
Rahi et al, 2006[17]	One Urban slum in Delhi, Births recorded during April-June 2005, cross sectional survey	n= 82 births<br><br>Home births= 56.1%	Not reported
Agarwal[18] et al, 2007	One urban slum in Delhi, Women who delivered last 1 year, Cross sectional survey	n= 82<br>Home births = 31.8%	Lack of awareness for need for check-up (27%)<br>Lack of knowledge about service availability (17%)<br>Long waiting time (22%)<br>None to accompany (15%)<br>Finance (12%)<br>Fear of hospitals (7%)<br>Family objections (2%)
DLHS Fact sheet (2007-8)[9]	Delhi state in 2008 using multistage stratified probability sampling	n= 9689 households<br>Home births<br>Rural = 42.6%<br>Urban = 29.9%<br>Total = 30.8%	Not reported
Thind et al, 2008[15]	NFHS survey data from Maharashtra , cross sectional survey	n=1510 recent births<br>Home births(overall) = 37%<br>Only Urban = 15.3%	Predisposing factors Religion(Hindu), multiple births and caste
Agarwal S et al , 2010[19]	11 slums of Indore, MP, Cross sectional survey of mothers of infants(2004-06)	n= 312<br>Home births= 56.4%	Not reported
Das S, 2011[20]	Mumbai slums from 6 municipal wards, survelllance study (2005-2007)	n= 10,754 births<br>Home births =10%	Customary(28%) , No time to reach hospital(13%), no body to go along(8%), Fear(7%)
Dasgupta et al, 2006[16]	Rural and urban clusters in West Bengal from Birbhum district. Cross sectional survey, women	n= 320<br>Home births (rural and urban combined) =51.88%	Not reported

For peer review only - http://bmjopen.bmj.com/site/about/guidelines.xhtml

	who delivered in the last one year		
Khan Z et al, 2009 [21]	Periurban area of Aligarh, Uttar Pradesh	n=92 mother of infants<br>Home births = 60%	Tradition (42%)<br>Related to economics(31%)
Hazarika, 2009[8]	NFHS- 3 Delhi data, Cross sectional survey, women who delivered 6 months ago	n= 2420 (slum dwellers)<br>Home births= 22.62%	Not reported

Qualitative data from our study suggest that there was a lack of perceived risk among women and their family members. According to the mothers and the family members who played an important role in decision making, the practice of giving birth at home was common and that they had witnessed their relatives doing well after doing the same. Priority was given to other domestic responsibilities and tradition over safe births. This may have been particularly relevant among multiparous women.

A low literacy level of the HOH was another important predictor indicating the need to raise awareness of safe birthing practices. The role of CHWs in improving maternal and neonatal health indicators by increasing awareness is well known.[28] Though the Delhi State Health Mission has deployed Accredited Social Health Activists (ASHA) in the urban areas replicating the rural model, a recent evaluation has shown several implementation gaps.[29]

Afsana et al from Bangladesh report that cost, fear of hospitals due to lack of privacy, unfamiliar surroundings and stigma attached to hospital delivery were key reasons for women to choose home births.[30] While women in our study spoke about fear and embarrassment as deterrents, direct costs associated with delivery and stigma attached to a hospital delivery were not mentioned as factors affecting their choice. Traditionally, Indian women gave birth at home surrounded by close family members. There is evidence to show that the support of a female

For peer review only - http://bmjopen.bmj.com/site/about/guidelines.xhtml

relative during the birth process is beneficial and leads to better birth outcomes.[31] The state of Tamil Nadu has implemented a successful birth companion scheme through its public health system which addresses the issues of social support, fear and embarrassment.[32] This model provides a prototype that may be replicated, with suitable context-specific modifications, to address this important barrier to institutional deliveries.

In our study most births at home were conducted by Dai who lacked professional training in safe birthing practices. An extensive review by Bergstrom and Goodburn has shown that traditional birth attendants had no impact on any reduction in maternal mortality[33]. A meta-analysis of training birth attendants improved survival but the studies included in the review were from high mortality burden rural population and not urban population. [34]Koblinsky et al[6] analysed national level data from several countries which reduced the MMR drastically since 1950 and showed that maternal deaths could be reduced by providing training to *dais or* professionals developing a partnership with *dais*, however it required apart from political will, effective outreach and referral mechanisms that support traditional system of birthing. Experiences from Malaysia and Sri Lanka show that, women are willing to move from home based to facility-based care if transport and services are made free for all, improved awareness and also ensured quality of services at facilities.[6] In India, currently there is no program at national scale for promoting or scaling up community based SBA. The National Rural Health Mission of Government of India has its main strategy for reduction in maternal mortality focused on facility based intra-partum care and provision of Emergency Obstetrics Care .

Current initiatives by the government aimed at improving MCH indicators of the urban poor do not directly address some of the key elements identified in our study. Identification and

For peer review only - http://bmjopen.bmj.com/site/about/guidelines.xhtml

mapping of the most vulnerable populations within the city, sensitization of health professionals to the needs and fears of women, improving reach of CHW to the marginalized, empowering women with information regarding their healthcare entitlements, provision of BPL, ration and RSBY cards to the neediest are some of the key issues that need focused and aggressive implementation.

It is important for health departments to strengthen the supply side, be more accessible to those who need them the most and establish faith among the community. India needs to explore innovative ways at all levels of care to make birthing practices safer. There is hope that the urban health situation will improve in the coming years with the NUHM, if we intervene at the individual, community, system and policy level. The ANCHUL project and similar such endeavours all over the country will feed to provide innovative scalable strategies for the betterment of our urban community.

For peer review only - http://bmjopen.bmj.com/site/about/guidelines.xhtml

Acknowledgements: We would like to acknowledge all the families who volunteered to provide data for this survey. We thank the efforts of field staff (Field coordinators, Field supervisor, and Field interviewers), Data manager and Data entry operators.

Funding: WHO**,** Geneva (Alliance for Health Policy and Systems Research (HSS/AHPSR), The Special Programme for Research and Training in Tropical Diseases (IER/TDR), The Special Programme of Research, Development and Research Training in Human Reproduction (HRP), Child and Adolescent Health and Development (FCH/CAH)) (Grant 2011/139172-0). The funders did not have any role in study design; the collection, analysis, and interpretation of data; the writing of the article or the decision to submit this article for publication

Contributors: SGJ and ND conceived and designed the study with additional inputs from AHS and SZ. ND analysed the quantitative data and prepared the first draft of the manuscript. SMG analysed the qualitative data and wrote the first draft of the qualitative findings. GA, and AHS supervised data collection and commented on drafts of the manuscript. HN and AHS commented on drafts of the manuscript. SZ is the Principal Investigator of ANCHUL, had overall responsibility for ANCHUL, and commented on drafts of the manuscript. All authors contributed to critique and modification of the manuscript, read and approved the final version. ND had full access to all the data in the study and had final responsibility for the decision to submit for publication.

Competing Interests: None

Data sharing statement: All unpublished data related to this research project is available with the authors and can be requested by emailing to niveditha@iiphd.org.

For peer review only - http://bmjopen.bmj.com/site/about/guidelines.xhtml

For peer review only

Ethics Approval: The study protocol was approved by Health Ministry Screening Committee of the Government of India, Institutional ethics committees of the Public Health Foundation of India, All India Institute of Medical Sciences, WHO Geneva and Harvard School of Public Health.

Legends:

Figure 1: Quantitative survey sampling

Figure 2: Conceptual framework of factors leading to home births among urban poor

For peer review only - http://bmjopen.bmj.com/site/about/guidelines.xhtml

REFERENCES

1. Ronsmans C, Graham WJ, Lancet Maternal Survival Series steering g. Maternal mortality: who, when, where, and why. Lancet 2006;**368**(9542):1189-200 doi: 10.1016/S0140-6736(06)69380-X[published Online First: Epub Date]|.

2. Montgomery AL, Ram U, Kumar R, et al. Maternal mortality in India: causes and healthcare service use based on a nationally representative survey. PLoS One 2014;**9**(1):e83331 doi: 10.1371/journal.pone.0083331[published Online First: Epub Date]|.

3. Koblinsky M, Matthews Z, Hussein J, et al. Going to scale with professional skilled care. Lancet 2006;**368**(9544):1377-86 doi: 10.1016/S0140-6736(06)69382-3[published Online First: Epub Date]|.

4. Campbell OM, Graham WJ, Lancet Maternal Survival Series steering g. Strategies for reducing maternal mortality: getting on with what works. Lancet 2006;**368**(9543):1284-99 doi: 10.1016/S0140-6736(06)69381-1[published Online First: Epub Date]|.

5. Ronsmans C, Scott S, Qomariyah SN, et al. Professional assistance during birth and maternal mortality in two Indonesian districts. Bull World Health Organ 2009;**87**(6):416-23

6. Koblinsky M, Kureshy N. Safe motherhood case studies: learning with stakeholders in South Asia--an introduction. J Health Popul Nutr 2009;**27**(2):89-92

7. WHO,UNICEF,UNFPA and The World Bank estimates: Trends in Maternal Mortality: 1990 to 2010, http://whqlibdoc.who.int/publications/2012/9789241503631_eng.pdf, (Accessed on 30th Decemebr, 2012).

8. Available from http://www.unescap.org/stat/apex/2/apex2_s.2_india_mdgs.pdf (accessed on 1st June, 2012).

9. Women and health: Health service coverage data by country: http://apps.who.int/gho/data/node.main.223, (Accessed December 30th, 2012).

10. Janani Suraksha Yojna. http://www.mohfw.nic.in/NRHM.htm (accessed on Jan 2013).

11. Lim SS, Dandona L, Hoisington JA, et al. India's Janani Suraksha Yojana, a conditional cash transfer programme to increase births in health facilities: an impact evaluation. Lancet 2010;**375**(9730):2009-23 doi: 10.1016/S0140-6736(10)60744-1[published Online First: Epub Date]|.

For peer review only - http://bmjopen.bmj.com/site/about/guidelines.xhtml

12. Randive B, Diwan V, De Costa A. India's Conditional Cash Transfer Programme (the JSY) to Promote Institutional Birth: Is There an Association between Institutional Birth Proportion and Maternal Mortality? PLoS One 2013;**8**(6):e67452 doi: 10.1371/journal.pone.0067452[published Online First: Epub Date]|.

13. Hazarika I. Women's reproductive health in slum populations in India: evidence from NFHS-3. Journal of urban health : bulletin of the New York Academy of Medicine 2010;**87**(2):264-77 doi: 10.1007/s11524-009-9421-0[published Online First: Epub Date]|.

14. District Level Household and Facility Survey-Factsheet, Delhi, Available at http://www.rchiips.org/pdf/rch3/state/Delhi.pdf.

15. National Urban Health Mission, http://www.uhrc.in/module-ContentExpress-display-ceid-95.html (Accessed on Dec 31, 2012).

16. Available at http://pipnrhm-mohfw.nic.in/index_files/non_high_focus_small/Delhi/Executive_Summary_2011-12_final.pdf.

17. NCR policy zones, Demographic profile and Settlement pattern (2001) Available at http://ncrpb.nic.in/pdf_files/SG1.pdf.

18. JJ cluster list; Available from http://delhishelterboard.in/main/. As accessed on 11th July,2012.

19. Vyas S, Kumaranayake L. Constructing socio-economic status indices: how to use principal components analysis. Health policy and planning 2006;**21**(6):459-68 doi: 10.1093/heapol/czl029[published Online First: Epub Date]|.

20. Thind A, Mohani A, Banerjee K, et al. Where to deliver? Analysis of choice of delivery location from a national survey in India. BMC Public Health 2008;**8**:29 doi: 10.1186/1471-2458-8-29[published Online First: Epub Date]|.

21. Dasgupta S, Das P, Mandal NK, et al. A study on intranatal care practices in a district of West Bengal. Indian J Public Health 2006;**50**(1):15-8

22. Rahi M, Taneja DK, Misra A, et al. Newborn care practices in an urban slum of Delhi. Indian J Med Sci 2006;**60**(12):506-13

23. Agarwal P SM, Garg S. Maternal health care utilization among women in an urban slum in Delhi. Indian Journal of Community Medicine 2007;**32**(3):203-05

For peer review only - http://bmjopen.bmj.com/site/about/guidelines.xhtml

For peer review only

24. Agarwal S, Sethi V, Srivastava K, et al. Birth preparedness and complication readiness among slum women in Indore city, India. J Health Popul Nutr 2010;**28**(4):383-91

25. Das S, Bapat U, More NS, et al. Prospective study of determinants and costs of home births in Mumbai slums. BMC Pregnancy Childbirth 2010;**10**:38 doi: 10.1186/1471-2393-10-38[published Online First: Epub Date]|.

26. Khan Z, Mehnaz S, Khalique N, et al. Poor perinatal care practices in urban slums: possible role of social mobilization networks Indian J Community Med 2009;**34**(2):102-07

27. Singh PK, Rai RK, Singh L. Examining the effect of household wealth and migration status on safe delivery care in urban India, 1992-2006. PLoS One 2012;**7**(9):e44901 doi: 10.1371/journal.pone.0044901[published Online First: Epub Date]|.

28. Lassi ZS, Haider BA, Bhutta ZA. Community-based intervention packages for reducing maternal and neonatal morbidity and mortality and improving neonatal outcomes. Cochrane Database Syst Rev 2010(11):CD007754 doi: 10.1002/14651858.CD007754.pub2[published Online First: Epub Date]|.

29. Sixth Common Review Mission Report. Available at http://nhsrcindia.org/pdf_files/resources_thematic/Health_Sector_Overview/NHSRC_Contribution/Final_6thCRM_Book_NRHM.pdf. Accessed on May 2013.

30. Afsana K, Rashid SF. The challenges of meeting rural Bangladeshi women's needs in delivery care. Reprod Health Matters 2001;**9**(18):79-89

31. Hodnett ED, Gates S, Hofmeyr GJ, et al. Continuous support for women during childbirth. Cochrane Database Syst Rev 2012;**10**:CD003766 doi: 10.1002/14651858.CD003766.pub4[published Online First: Epub Date]|.

32. Padmanaban P, Raman PS, Mavalankar DV. Innovations and challenges in reducing maternal mortality in Tamil Nadu, India. J Health Popul Nutr 2009;**27**(2):202-19

33. Bergstrom S GE. The role of traditional birth attendants in the reduction of maternal mortality. Stud Health Serv Organ Policy 2001;**17**:77-96

34. Wilson A, Gallos ID, Plana N, et al. Effectiveness of strategies incorporating training and support of traditional birth attendants on perinatal and maternal mortality: meta-analysis. BMJ 2011;**343**:d7102 doi: 10.1136/bmj.d7102[published Online First: Epub Date]|.

For peer review only - http://bmjopen.bmj.com/site/about/guidelines.xhtml

Why ~~do~~ women ~~deliver at~~choose -to give birth at home:~~?~~ A situational analysis from urban slums of Delhi

Niveditha Devasenapathy[1], MBBS, M.Sc, Mathew Sunil George[1], MPH, Suparna Ghosh Jerath[1], PhD, Archna Singh[2], PhD, Himanshu Negandhi[1], MD, Gursimran Alagh B.Sc[1], Anuraj H Shankar[3], D.Sc, Sanjay Zodpey[1] MD, PhD

[1] Indian Institute of Public Health, Delhi, Public Health Foundation of India, Gurgaon, India,
[2]Department of Biochemistry, All India Institute of Medical Sciences, New Delhi, India
[3]Department of Nutrition, Harvard School of Public Health, Boston, Massachusetts, USA

Address for correspondence

Dr Niveditha Devasenapathy MBBS, MSc
~~Assistant~~ Associate Professor
Indian Institute of Public Health- Delhi
Plot No. 34, Sector 44, Institutional Area, Gurgaon 122002, India
Phone: 91-124-4722922, Fax : 91-124-4722901
email:niveditha@iiphd.org

Key Words: Reproductive Health, Access to Health Care, Health Promotion, Needs Assessment, Maternal Health

Word count
Abstract: 273
Main text: : ~~3596~~4128

For peer review only - http://bmjopen.bmj.com/site/about/guidelines.xhtml

ABSTRACT

~~Background:~~Objectives: Increasing ~~I~~institutional births ~~delivery~~ is an important strategy for ~~towards~~ attaining MDG-5. However, ~~R~~rapid growth of low-income and migrant populations in urban settings in low and middle income countries, including India, ~~India and other countries has placed~~ presents unique ~~additional demands on government~~ challenges for programs ~~to enhance~~ to improve utilization of institutional care. Better understanding of the factors influencing home or institutional birth ~~barriers to institutional deliveries~~ among the urban poor is urgently needed to enhance program impact. ~~to enhance program impact in India, and elsewhere.~~

~~Objectives:~~ To measure prevalence of home and institutional ~~deliveries~~ births in an urban slum population ~~in Delhi, India, slums~~ and identify factors influencing these events. ~~the reasons for home delivery~~births.

~~Study d~~Design: Cross-sectional survey using quantitative and qualitative methods

Setting: Urban poor settlements in Delhi, India

~~Methods~~Participants: A house-to-house survey was conducted of all households in three slum-clusters in north-east Delhi (n=32,034 individuals). Data on ~~deliveries~~ birthing place and socio-demographic characteristics were collected using structured questionnaires (n=6092 households). Detailed information on pregnancy and postnatal care was obtained from women who ~~had delivered~~gave birth in the last 3 months (n=160). Focus group discussions and in-depth interviews were conducted with stakeholders from the community and health-care facilities.

For peer review only

~~Risk-factors for place of birth home delivery were examined using random effects logistic regression.~~

Results: Of 824 women who gave birth~~delivered~~ in the previous year, 53% [95%CI 49.7-56.6] had given ~~ave~~ birth at home.~~delivered at home~~ ~~.~~In adjusted analyses, multiparity, low literacy and migrant status were independently predictive of home ~~delivery~~births. Fear of hospitals (36%), comfort of home (20.7%) and lack of social support for child-care (12.2%) emerged as the primary reasons for home ~~delivery~~births.

Conclusions: Home ~~deliveries~~ births are frequent among the urban poor. ~~In addition to current financial initiatives for institutional delivery,~~ This study highlights the urgent need for ~~raising community awareness and~~ improvements in the quality and hospitality of client services, ~~and~~ needs for family support ~~are~~ as key modifiable factors affecting over two thirds of this population. ~~identified in this study.~~ These findings should inform the design of strategies to promote institutional births ~~delivery~~.

For peer review only

Article Summary

Strengths and Limitations of the study

- This survey covered a large number of households (n= 6092) households living in 3 urban poor settlements of Delhi
- ~~and b~~Both qualitative and quantitative methods were used to capture reasons for home ~~delivery~~births.
- Though the slum cluster was not a random sample from all the slum clusters in Delhi they were representative of the urban poor settlements.
- Concurrent health facility assessment was not done which would have helped to understand additional ~~the~~ supply~~-~~side factors~~issues~~.

For peer review only - http://bmjopen.bmj.com/site/about/guidelines.xhtml

~~BACKGROUND~~ INTRODUCTION

Increasing ~~I~~institutional births ~~delivery~~ is ~~currently~~ a key global strategy to reduce maternal and newborn mortality. Many countries, including India, have established incentive programs and policies to enhance institutional births~~deliveries~~. However, the rapid growth of low-income urban populations presents unique challenges to these programs such as lower knowledge of local services and registration processes, lack of support from an extended family and transient residence. With around 40% of ~~persons~~urban population in low and middle income countries residing in low-income urban settlements[1], ~~, and~~ more focused ~~targeted~~ efforts are ~~may be~~ required to improve institutional birth ~~delivery~~ rates in these ~~low-income urban~~ settings.

Most maternal deaths are centred around the intra-partum and immediate postpartum period [1 2] and for countries with high burden of maternal mortality and morbidity, facility based birthing is found to be efficient and sustainable compared to scaling up community based safe birthing programs.[3 4] [6]An evaluation study of the safe motherhood program in Indonesia showed that irrespective of level of socioeconomic status and place of residence (urban vs. rural) increasing the number of deliveries by Skilled Birth Attendant (SBA) did not reduce maternal mortality if most births took place at home. [5] In contrast, programs that enhanced facility-based births in Malaysia and Sri Lanka resulted in marked reduction in maternal and neonatal deaths.[6] With the overwhelming evidence for scaling up quality health centre based intra-partum care in improving maternal and neonatal survival, it is important for each country and local administration to understand barriers at community and facility level that affect the access to facilities and provision of quality services.

For peer review only

~~-~~India currently accounts for about a fifth of all maternal and ~~newborn~~new-born deaths worldwide ~~.~~[7], and approximately one third of the population currently lives in urban areas, and growing to nearly one half by 2030. The MDG report has flagged the slow progress of India in reducing child mortality and improving maternal health ~~.~~[8], with the latest WHO statistics showing that only half of expectant mothers in India complete four antenatal care (ANC) visits and give birth in the presence of a ~~skilled birth attendant (SBA~~SBA. ~~)~~[9]~~,~~ Overcoming barriers to institutional births among the urban poor is therefore crucial.

~~The choice of place of delivery~~for birthing ~~has been driven by tradition, accessibility and economics. In India, MMR is high in states with high prevalence of home deliveries~~births~~.~~ The National Rural Health Mission (NRHM) of India therefore launched the Janani Suraksha Yojana (JSY) program in 2005 ~~a safe motherhood intervention under the National Rural Health Mission (NRHM)was implemented in India in 2005~~ with the goal ~~objective~~ of reducing maternal and neonatal mortality by promoting institutional births ~~delivery~~ among poor pregnant women.[10] An evaluation of this conditional cash transfer scheme in 2007-08 showed an increase in ANC visits and institutional births~~deliveries~~.[11] However, this ~~increase in institutional deliveries~~ has not translated to reduction in MMR possibly ~~probably~~ due to unaddressed issues of non-financial access barriers and sub-optimal ANC, delivery and postnatal care.[12] In addition, unique issues faced by urban poor were not specifically addressed by the JSY program.

For peer review only - http://bmjopen.bmj.com/site/about/guidelines.xhtml

As mentioned above, ~~C~~currently 3~~5~~0% of India population are living in cities. Delhi is one of the most densely populated cities in the world, and ~~. Delhi~~ attracts nearly 500,000 migrants every year with most ~~of who mostly~~ settlinge ~~down~~ in urban poor habitations.

According to the National Family Health Survey (NFHS 3) survey conducted in 2005-06, only 44 % of ~~deliveries~~ births were institutional among the urban poor of Delhi as compared to the urban average of 67.5%.[13] The District Level Household and Facility Survey (2007-08) survey showed that overall, 71% of pregnant women had at least 3 ANC visits. While 68% of ~~deliveries~~ births were institutional in the city as a whole, only 38% institutional deliveries were reported in slum areas.[14] A governmental initiative aimed at correcting this inequity is the National Urban Health Mission (NUHM) which makes ~~essential~~ primary health care services available to the urban poor.[15],[16] The success of this mission will depend on identifying and targeting interventions directed towards the most vulnerable. One of the aims of this study is to determine the prevalence of home and institutional ~~delivery~~ births among women living in urban poor settlements in Delhi and identify reasons influencing ~~behind~~ their choice ~~to deliver at~~for ~~home~~ births.

METHODS

This study ~~analysis~~ is part of the formative phase and situational analysis for the ANCHUL (Ante Natal and Child Health care in Urban Slums, CTRI/2011/091/000095) trial ~~study,~~ ~~which is~~ an implementation research project aimed to develop, implement and evaluate the impact ~~effectiveness~~ of an intervention package delivered through an urban community healthcare worker (UCHW). ANCHUL aims to increase access to ~~institutional deliveries~~health care facilities for birthing and improve maternal, neonatal, child healthcare (MNCH) practices in urban slums of Delhi. ~~The objective of t~~This ~~formative~~ study aimed ~~was~~ to conduct an in-depth situational

For peer review only - http://bmjopen.bmj.com/site/about/guidelines.xhtml

analysis on utilization and quality of MNCH care using quantitative and qualitative methods.

The information obtained from this study will guide the development of the community based intervention package to be delivered by the UCHW as part of the ANCHUL trial.

Study Setting

Of the 16.7 million people living in Delhi, 52% reside in poor habitations.[17] The north-east district of Delhi contributes to 11%, about one fifth, -to of thise total population with 44 registered slums.[18] This district has the highest home delivery births rates.[14] We conducted a rapid survey in 17 slum clusters to obtain information on number of households, water supply, sanitation, presence of schools, healthcare facility, and distance from nearest government hospital. The clusters were then stratified into two categories of vulnerability based on the above characteristics. We then randomly selected chose three vulnerable slum clusters namely Buland Masjid (BM), CPJ and Chanderpuri (CP). for the purpose of this situational analysis . These slums had metalled tarred roads within and had access to maternal child health care dispensaries within a distance of five kms. The study protocol was approved by Health Ministry Screening Committee of the Government of India, institutional ethics committees of the Public Health Foundation of India, All India Institute of Medical Sciences, WHO Geneva, and Harvard School of Public Health.

Data collection

Quantitative survey

After lane mapping the clusters, all households were included in the survey. We identified pregnant women (in their 2nd and 3rd trimesters), recently delivered women (RDW, i.e., those who had delivered in the last 3 months) and households with under-5 children. The purpose of

For peer review only - http://bmjopen.bmj.com/site/about/guidelines.xhtml

the survey was explained to a household member above 18 years of age and all questionnaires were administered after obtaining informed consent by trained field interviewers. All survey tools were in local language and were piloted and modified for content and clarity. Information on family details, socio-demographic status, place of ~~delivery~~ childbirth (in women who had ~~delivered~~ given birth in past 1 year), and information on any maternal and child deaths within households in past 1 year, was obtained using paper forms. All refusals and non-responses were documented. We re-visited the households of 160 RDW and collected detailed information about ANC, delivery, immediate post-natal period, new born care practices and diet of the mother. Data were checked for completion before entering into a ~~validated~~ structured database management system (Microsoft ~~a~~Access 2010) with inbuilt range and internal consistency checks. Information from RDW was edited and validated by double data entry.

Qualitative data

The categories of respondents in *table 1* were identified as relevant for data collection in this study. Households were informed that focus group discussions (FGDs) would be held in the community and a general invitation was given. Permissions were sought from local community and religious leaders. Local public and private health care providers were approached and permissions were sought for in-depth interviews (IDI). The FGD and IDI guides were piloted to refine the topic guides to enable them to generate data that was relevant to the study objectives. The main topics that were explored in the FGDs and interviews are shown in *table 1*. The venue for data collection was agreed upon based on the respondent's convenience. One interviewer facilitated the discussions while a second took notes. Based on responses from the community, health care facilities and traditional birth attendants who served the locality were

For peer review only - http://bmjopen.bmj.com/site/about/guidelines.xhtml

identified and approached. Written informed consent was obtained from all participants before the FGDs and IDIs, which were digitally voice-recorded.

Sample size justification

For estimating the number of households to be interviewed, institutional birth ~~delivery~~ was considered the key outcome variable. Assuming prevalence of institutional ~~deliveries~~ births as 33% in urban slums of Delhi[16], we needed to interview 780 women who gave birth ~~delivered~~ in the past ~~one~~ year to obtain ~~current~~ prevalence estimates with 10% relative precision. Assuming a crude birth rate of 25/1000 (national average is 21/1000), a population of 30,000 was to be covered to identify at least 750 ~~deliveries~~ childbirths ~~that happened~~ in the past ~~one~~ year.

Table 1: Themes covered for qualitative data

Category of participants	Method of data collection	Key themes covered
**Community**<br>• Pregnant women (n= 5)<br>• Recently delivered women (n=6)<br>• Mother of under 5 children (n=6)<br>• Mother in laws (n=5)<br>• Husbands (n=4)	Focus Group Discussions(FGDs)<br><br>Venue: Schools, NGO, Madrassa(religious place) and anganwadi centres*	• Health and nutritional status<br>• Cultural practices for nutrition during pregnancy<br>• Care seeking behaviour during pregnancy<br>• Barriers to accessing care during pregnancy<br>• Quality of care experienced in various health care settings (public and private)
**Health Care Providers**<br>• Public Health system (n=6)<br>• Private (n=5)<br>• Others (n=4) (AWW, TBAs)	In depth interviews<br>Venue: Clinics of health care providers or homes of key informants	• Care seeking pattern among the community during pregnancy<br>• Challenges to improving maternal and child health among urban poor<br>• Feasibility of proposed intervention
**ANC Clinic attendees** (n=9)	Exit interviews (pre and post ANC check-ups)<br>Venue: Clinics	• Experience of care during ANC visit<br>• Satisfaction levels of the individual about care

For peer review only - http://bmjopen.bmj.com/site/about/guidelines.xhtml

AWW: Anganwadi workers, TBA: Traditional birth attendants *(Dai)*
*****The word Anganwadi means "courtyard shelter" in Hindi. They were started by the Indian government in 1975 as part of the Integrated Child Development Services program to combat child hunger and malnutrition.

Data analysis

Quantitative data

Data were analysed using Stata 11 (Stata Corporation, College Station, TX). Descriptive statistics were used to provide a cluster, household and individual level profiles of the study population. Household survey data were analysed accounting for clustering at the slum level to control for both inter and intra-cluster variance. We used principal component analysis to compute household Socioeconomic Scales (SES). Dwelling characteristics, household income and household assets were included in this composite scale.[19] We used multi~~tli-~~variate~~ble~~ random effects logistic regression to estimate the association of demographic variables with home births~~-delivery~~. Crude and adjusted odds ratios were calculated with 95% confidence intervals. For data from RDWs, Pearson chi-squared was used for categorical variables and t-tests for comparison of continuous variables.

Qualitative data

Verbatim transcripts were prepared in a standardized format that included basic demographic information of the participants and the interviewer's own observations within one week of conduct of IDI/FGD. Transcripts were uploaded to a software Atlas ti 6.1 (Scientific Software Development, City West, Berlin) and coded line-by-line using detailed themes and sub-themes that emerged from the data. After an initial round of coding with a representative

For peer review only - http://bmjopen.bmj.com/site/about/guidelines.xhtml
For peer review only

sample of transcripts, the list of codes that were generated was reviewed in order to develop a structured code list which was then applied to the remaining transcripts. Illustrative quotations that captured the key issues reported by the participants from the transcripts have been included in the results.

RESULTS

Of the 6348 households in the three defined clusters, 6092 (96%) households were interviewed between December 2011 and March 2012, covering a total population of 32,034. Nine households refused to participate and 247 households did not respond (locked houses) (figure 1). A total of 25 FGDs and 13 in-depth interviews were conducted in January and February 2012. The number of respondents in each FGD ranged from 7-12 members.

Population and cluster characteristics

The adult male to female ratio was 1000:825. Fifty eight percent of the population were migrants from Uttar Pradesh (73%) and Bihar (16%). Eighty percent of the population were living in the same locality for > 5 years. Of the total population, women of in reproductive age (15-49 years) accounted for 25%, and 16.6% were under-5 children. The area was served by one referral hospital situated within a distance of 5kms, two outpatient dispensaries, 17 private clinics (registered and unregistered with the Medical Council) and one laboratory within the clusters. The areas also have access to two referral hospitals situated at distance of about 10 kms.

For peer review only - http://bmjopen.bmj.com/site/about/guidelines.xhtml

Household characteristics

The median family size was 5 (IQR 4, 7) predominantly living as nuclear families (79.4%) and 63% of houses were self-owned. The head of the household (HOH) was the one considered as the decision--maker but was not necessarily the primary wage earner~~ of the family~~. Fifty nine percent of HOHs were illiterate and were unskilled labourers. Ration cards, Below Poverty Line cards (BPL) and Rashtriya Swasthya Bima Yojna (RSBY) cards that are needed for claiming government run health schemes were possessed ~~only~~ by only 50%, 31% and 24% of households respectively. The ~~M~~majority of the households (82%) lived in single-roomed concrete houses ~~and~~ with cemented or tiled flooring. Most houses (95%) had access to toilets~~,~~ within the household or community. A detailed socio-demographic profile of the study population is presented in *Table 2*. The household characteristics of the ~~subsample of~~ RDW and the overall population in the study area were similar indicating that our subsample households were representative of this area. Fifteen maternal deaths, 21 still-births and 41 under-5 child deaths were reported for the previous year. Of the total child deaths, 22 were in the neonatal period.

~~**Figure 1: Quantitative survey sampling**~~

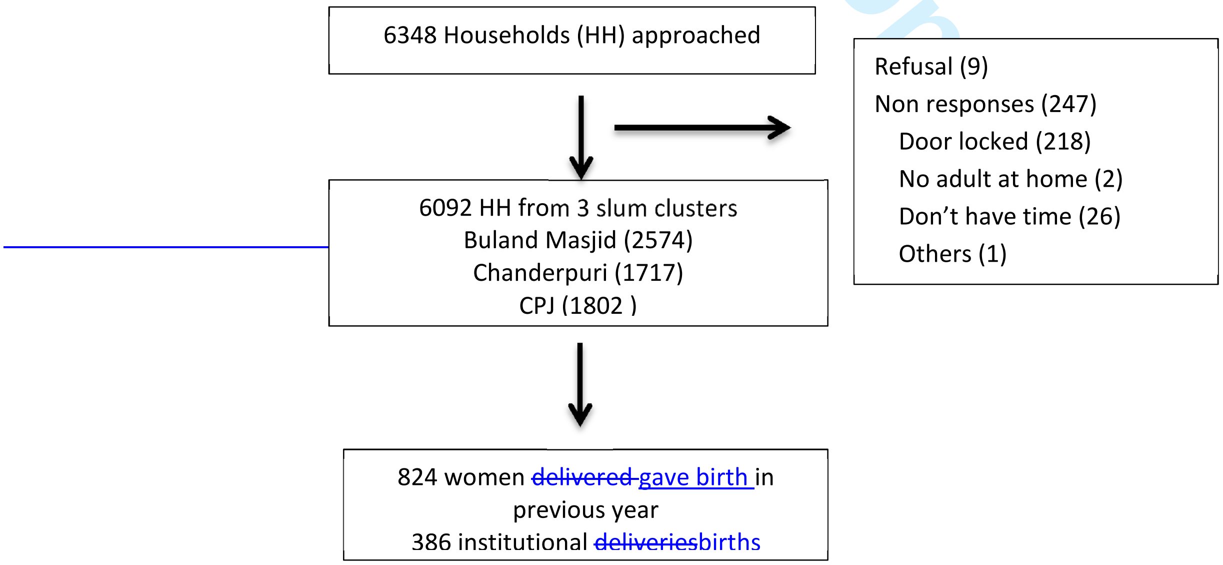

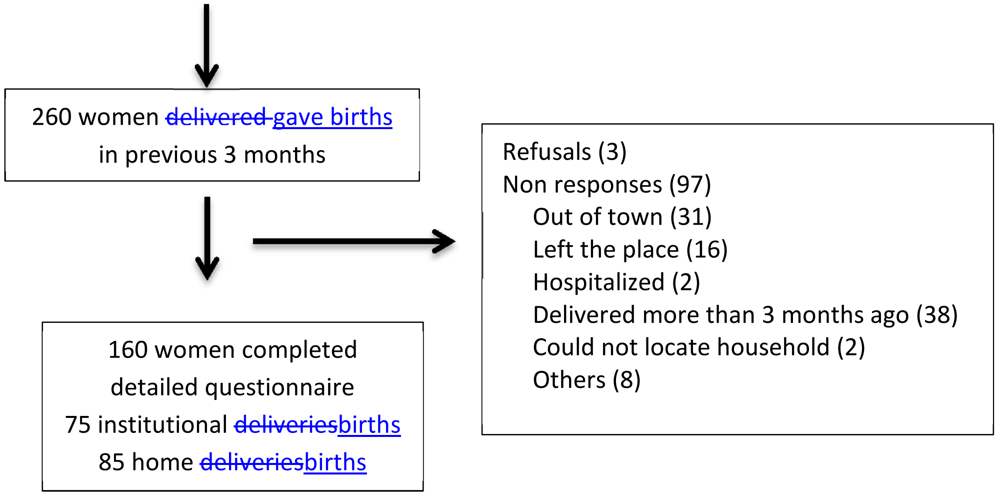

For peer review only

For peer review only - http://bmjopen.bmj.com/site/about/guidelines.xhtml

Table 2: Characteristics of households in the study area and households of women who delivered gave birth in previous 3 months

Demographic characteristics	House to House survey (n=6092 HH from 3 clusters	Recently DeliveredMothers who recently gave birth Women (n=160)
Median HH size (IQR)	5(4,7)	5 (4,7)
Family type (%)		
Nuclear	4834(79.4)	115 (71.9)
Joint	944(15.5)	43 (26.9)
Extended	313 (5.1)	2 (1.3)
Spoken language (%)		
Hindi	5328 (87.5)	145 (90.6)
Urdu	624(10.2)	15 (9.4)
Others	140(3.2)	-
Religion (%)		
Hindu	1822(29.9)	42 (26.3)
Muslim	2475 (69.6)	118 (73.8)
Others	33(0.5)	-
Caste category (%)		
General	2546 (41.8)	77 (48.1)
Other Backward Caste	2553 (41.9)	58 (36.3)
Scheduled caste/Scheduled Tribe	932 (15.3)	22 (13.8)
Do not want to answer	4 (0.1)	-
Do not know	57 (0.9)	3 (1.9)
Illiterate women in reproductive age group (%)	(n= 8056) 4122(51.2)	85 (53.1)
Literacy level of HOH (%)		
Illiterate	3561(58.5)	93 (58.1)
Literate but no formal education	196(3.2)	4 (2.5)
Schooling	2129(36.4)	62 (38.8)
College	115(1.89)	1 (0.6)
Occupation of HOH (%)		
Unskilled	2805 (46.3)	77 (48.4)
Skilled	1378 (22.7)	36 (22.6)
Office work	867 (14.3)	19 (12)
Professional	55 (0.91)	-
Not working	955 (15.76)	27 (17)
Median HH income in INR (IQR)	4000(3000-6500)	4000 (3000-7000)
Median HH income in USD (IQR)	76.2(57.1- 123.8)	76.2 (57.1-133.5)
Own house (%)	3829 (62.9)	101 (63.1)
Ration card (%)		
Don't have	2994 (49.2)	91 (56.9)
White	1173 (19.3)	22 (13.8)
Yellow	1196 (19.6)	28 (17.5)
Pink	686 (11.3)	18 (11.3)
Do not want to answer	43 (0.7)	1 (0.6)

For peer review only - http://bmjopen.bmj.com/site/about/guidelines.xhtml

BPL card (%)	1903 (31.2)	47 (29.4)
RSBY card (%)	1461 (24)	36 (22.5)
% of HH who are staying in the current locality in years		
< 1	660 (10.8)	13 (8.1)
1-2	238 (3.9)	11 (6.9)
3-5	330 (5.4)	12 (7.5)
>5	4864 (79.8)	124 (77.5)
% belonging to Delhi	3572(57.49)	93 (58.1)
% of HH who migrated but living in Delhi		
< 1 year	261 (7.5)	2 (2.2)
1-2	123 (3.5)	3 (3.2)
3-5 years	195 (5.6)	2 (2.2)
>5 years	2920 (83.5)	86 (92.5)
~~**Living conditions**~~		
~~Concrete houses (%)~~	~~4826 (79.2)~~	~~125 (78.1)~~
~~Cemented floor (%)~~	~~5829 (95.7)~~	~~153 (95.6)~~
~~Houses with only one room~~	~~4978 (81.7)~~	~~124 (77.5)~~
~~% of HH with separate kitchen~~	~~1168 (19.2)~~	~~33 (20.6)~~
~~Fuel used for cooking (%)~~		
~~Gas~~	~~3511 (57.6)~~	~~101 (63.1)~~
~~Kerosene~~	~~1831 (30.1)~~	~~44 (27.5)~~
~~Firewood~~	~~572 (9.4)~~	~~13 (8.1)~~
~~Electricity~~	~~92 (1.5)~~	~~2 (1.2)~~
~~Do not cook at home~~	~~86 (1.4)~~	~~-~~
~~Public source of drinking water (%) (handpump/ tanker/bore)~~	~~5102 (83.6)~~	~~132 (82.5)~~
~~Defecation facilities (%)~~		
~~Toilet within house~~	~~4571 (75.1)~~	~~125 (78.1)~~
~~Community/shared~~	~~1242 (20.4)~~	~~30 (18.8)~~
~~Defecate in open~~	~~278 (4.6)~~	~~5 (3.1)~~
Socioeconomic categories* (%)		
Lowest	1.976(32.45)	53 (33.1)
Middle	2,077 (34.11)	53 (33.1)
Highest	2,036 (33.44)	54 (33.8)
	~~4350 (71.4)~~	~~112 (70)~~
	~~1557 (25.6)~~	~~46 (28.8)~~
	~~1054 (17.3)~~	~~29 (18.1)~~
	~~4403 (72.3)~~	~~115 (71.9)~~
Distance of HH from nearest Maternal Child Health care centre		
<5 km	4882 (80.1)	125 (78.1)
5-10 km	1550 (18.9)	29 (18.1)
>10 km	60 (1)	6 (3.8)

For peer review only

HOH: Head of Houshold, BPL: Below Poverty Line card, RSBY: Rashtriya Swasthya Bhima Yojna(Health insurance scheme)*The scale is a composite of house type, floor, house ownership, separate kitchen, TV, Refrigerator, Mobile phone, washing machine, total HH income, Number of rooms by principal component analysis

For peer review only

For peer review only - http://bmjopen.bmj.com/site/about/guidelines.xhtml

Place of ~~delivery~~ childbirth

Of the 824 women who gave birth in the previous year, 438 ~~delivered~~ were ~~at~~ home births [53.1%, 95%CI (49.1 - 56.6)] and of the remaining 386, 340 (88%) chose to give birth at a public hospital. Among the women who ~~delivered~~ gave birth in the previous 3 months (n=160), a similar proportion [53.1 %, 95% CI (45.0- 61.0)] ~~delivered at home~~gave birth at home. Only 16.2% (12) of these mothers' availed cash incentive through JSY scheme *(table 3)*. Thirty six (48%) went to hospital due to initiation of labour pains, 32% due to development of complications, and 6.7% reported the reason that they had crossed expected date of delivery. The individual who was most influential in the decision for delivering at a hospital was most often (48%) the women herself, followed by the husband (18.7%) or mother-in-law (17.3%). Irrespective of the place of delivery only 15% of these households were visited by a~~n~~ ~~UCHW~~ health worker within 48hrs of delivery and only 30% of women visited a health care facility after ~~delivery~~giving birth. Among those who gave birth in a facility~~Most~~ ~~(~~92%~~)~~ were satisfied with the services provided at the hospital ~~during delivery~~. ~~Compared to women who delivered at a hospital, w~~Women who ~~delivered~~ gave birth at home were more likely to be multiparous, less likely to avail ANC in a public hospital and visit a facility during the post-partum period *(table 3).*

Predictors of home ~~delivery~~ births

Among the 824 women who ~~delivered~~ gave birth in the previous year, the following demographic characteristics were significantly associated with ~~delivering at home~~home births: Living in a rented house, low SES, low literacy of HOH, HOH being an unskilled labourer, migrants and multi-parity. Multi-parity [OR 1.96, 95% CI (1.44, 2.69)], literacy status of HOH [OR

For peer review only - http://bmjopen.bmj.com/site/about/guidelines.xhtml

0.71, 95%CI (0.53 0.97)] and migrant status [1.46, 95% CI (1.08, 1.97)] remained strong independent predictors of home ~~delivery~~ births in multivariate ~~able~~ analysis *(Table 4)*.

Table 3: Information on~~f~~ ANC and births obtained from recently delivered women

**Characteristics***	**Home ~~deliveries~~ births (n=85)**	**Institutional ~~deliveries~~ births (n=75)**	**p value**
Mean age	24.7(4.31)	24.9(4.43)	0.79
>18yrs of age at marriage	72 (84.7)	64(85.3)	0.912
Mean family size	5.5(2.4)	5.5(2.3)	0.937
First child	15 (17.7)	26 (34.7)	**0.014**
Some ANC care	63 (74.1)	72 (96)	**<0.0001**
ANC at public hospital	41 (65)	61(84.7)	**< 0.0001**
ANC in first trimester	21 (33.33)	31 (43.1)	0.498
Some health problem during pregnancy	11 (17.5)	13 (18)	0.92
Satisfaction with ANC	46 (74.6)	65 (90.3)	0.046
Planned place of ~~deliveries~~birth	73 (86)	66 (90.4)	0.38
~~Delivery~~ Birth conducted by Doctor/ Nurse	15 (17.7)	74 (98.7)	**<0.0001**
Home visitation by ~~a~~ community health worker	14 (16.5)	11 (14.7)	0.75
Post-~~delivery~~ partum visit to hospital	3 (3.53)	16 (21.33)	**0.001**

*All continuous variables are expressed as mean and one standard deviation; all proportions are expressed as percentages

Table 4: Predictors of home ~~delivery~~ births

**Characteristics (n=824) 438: Home ~~delivery~~ births 386: Institutional delivery**	**p value (ignoring clustering)**	**Crude OR (95% CI), p value (accounted for clustering)**	**Adjusted* OR (95% CI), p value (LR test)**
Buland Masjid<br>CPJ<br>Chaderpuri	<0.001	1 **<0.001**<br>0.42(0.30, 0.59)<br>0.69 (0.49, 96)	
Birth order second and above	<0.001	2.12 (1.57, 2.87) **<0.001**	1.96 (1.44, 2.69) **<0.001**
Lower SES<br>Middle SES<br>Highest SES	0.033	1<br>0.90 (0.64, 1.26) **0.011**<br>0.70 (0.50 0.98)	0.96 (0.66, 1.406) 0.68<br>0.91 (0.60, 1.38)
Joint families	0.057	0.78 (0.57, 1.05) 0.102	
Non-Muslims	0.004	0.77 (0.54, 1.1) 0.15	0.78 (0.54, 1.14) 0.21
Schooling of HOH	0.007	0.74 (0.55, 0.99) **0.04**	0.71 (0.53 0.97) **0.031**
Not working		1 **0.063**	1 0.2

For peer review only - http://bmjopen.bmj.com/site/about/guidelines.xhtml

Elementary job<br>Skilled job	0.024	1.63(1.08, 2.45)<br>1.42 (0.92, 2.17)	1.54 (1, 2.38)<br>1.40 (0.89, 2.21)
Own the house	0.004	0.76 (0.56, 1.02) **0.07**	0.94 (0.65, 1.34**)** **0.72**
Ration card possession	0.037	0.80 (0.60, 1.07) 0.14	
Not belonging to Delhi	<0.001	1.61(1.21, 2.15) **0.001**	1.46 (1.08, 1.97) **0.013**

*Adjusted for house ownership, SES, literacy of HOH, Occupation of HOH, belonging to Delhi and birth order

Reasons for ~~delivering at home~~choosing home birth

For peer review only

The majority of home ~~deliveries~~ births were pre-planned and 75% of these women had availed some ANC at a facility. Eighty two percent of the home ~~deliveries~~ births were conducted by a traditional birth attendant (*Dai*). The results from the quantitative and qualitative data, showed a high level of concordance for the reasons for choosing ~~to deliver at~~ home births. Four major themes emerged as barriers to institutional ~~delivery~~births. Illustrative quotations from the transcripts are presented in *table 5*.

Table 5: Illustrative quotations ~~from the transcripts~~ for reasons for ~~delivering at~~ home birth

Fear and Embarrassment<br><br>*"They prefer home deliveries as in many cases the doctor does not behave well with them. As soon as they enter, they are separated from their families. The doctor does not communicate to the relative if there is any complication. They stay for 1-2 days in the labour room; the relatives are outside they do not know what is happening. It is very scary for them. Also because of all these reasons, they will come only if it is life threatening. Even then they might prefer to go to someone who is local, who is more patient friendly, private practitioner who are non-judgemental who behave properly. They give more one to one care. They might not be qualified but it is more natural and human."* ***Senior gynaecologist, Public health facility***<br><br>*"I'd prefer to have the baby at home. If you're in hospital, they don't even attempt to try for a natural birth. What's the use of having an operation if you can have a child the normal way? We get the check-ups done there, but we end up having the delivery at home".* ***Pregnant woman***<br><br>*"If you tell them to put you in a closed room to get a check - up, they tell you to just lie down and get it done right there. It's humiliating; you can't help but feel embarrassed. And if you don't feel embarrassed, other people around you will. They tell you that if you feel ashamed, go to a private hospital. It's a matter of dignity. There are men walking around as well, if a man catches a glimpse, it can create trouble at home".-****Pregnant woman***
Prior experience with hospitals

"I had gone recently with my sister to a hospital……when I went there to deliver my baby, they just kept telling me 'keep pushing, keep pushing…" I got so scared I just left. I've had three children at home; I can manage a fourth the same way". ***Recently delivered woman***

Other children

"I have small children. If I have to go to the hospital, I have to lock the house and take the children along. Then if she delivers in the hospital, she may be admitted for at least 2 or 3 days depending on the situation. Even if it is a normal delivery it is at least a 2 day stay. How do I manage in such situations? To avoid all this one hopes that if all is well it is better to deliver at home itself. We can all be at home and kids need not have to go anywhere. I can also go for work." ***Husband of pregnant woman***

Opportunity costs

"Most of them earn daily wages, so they do not want to come to the hospital. They feel one day will go so they will lose their pay also...So the delivery can be at home if baby is okay. Male member does not want to involve himself in all these things, either if there is an elder woman in the house or neighbourhood who conducts the delivery...Even at the hospital they do not want to stay they say that they have to go to work, or how will they earn for tomorrow's food because they are working on daily wages.." ***Senior gynaecologist, public health facility***

Fear and embarrassment

Fear and embarrassment associated with giving birth in hospitals ~~was~~ were reported as the most important reasons for ~~delivering~~ giving birth at home during FGDs and IDIs and was also reported as the key reason (35%) among the 85 RDW surveyed. Fear was due to being alone in unfamiliar surroundings and fear of surgical intervention. In addition to fear, women felt ~~that~~ it was embarrassing and uncomfortable for them to be in the presence of 'strangers' during a very vulnerable time. The lack of privacy coupled with the absence of any family member by their side was in stark contrast to the 'safe and reassuring environment' at their homes during the ~~child~~ birthing process.

Prior experience with hospitals

For peer review only

Prior experience of self, friends, neighbours or a family member played an important role in ~~the choice~~ choosing ~~to deliver at~~ home or ~~in~~ hospital births. Positive experiences reinforced the message that hospitals were a safe and welcoming place as opposed to negative experiences (such as, perceived improper care and rude behaviour of hospital staff). The health care providers interviewed indicated that high patient load at hospitals lead to lack of individual attention and inadequate care.

Domestic responsibilities

Being in an unfamiliar neighbourhood, the absence of extended family to help with childcare and traditional lack of involvement of men in childcare made women reluctant to leave children at home and get admitted to hospital. In the survey, 10% of those who had ~~delivered at~~ home births cited lack of help with childcare as a reason.

Opportunity costs

Though most services at the hospital were provided free of cost, opportunity costs in the form of lost wages for the earning member, cost of food for the family and travel, dissuaded some women from delivering in hospital. Although only 6% mentioned this as a reason in our survey, this emerged as a factor in the qualitative analysis. However, direct costs were not one of the reasons cited for not opting for hospital births.

DISCUSSION

For peer review only - http://bmjopen.bmj.com/site/about/guidelines.xhtml

Our study showed a high prevalence of home ~~deliveries~~ births conducted by ~~*dais*~~ *TBA* among the urban poor of north-east district of Delhi. Fear of surgical procedures, unfamiliarity with hospital surroundings, lack of help for childcare and loss of wages, were some of the reasons that drove women to choose home ~~delivery~~briths. Other predictors of home ~~delivery~~ births were low literacy, higher parity and migrant status. Concordance between results derived from qualitative and quantitative data lends greater credibility to these findings. *Figure 2* presents a conceptual framework based on the study findings which could help us to design strategies for some of the modifiable factors.

The prevalence and reasons for home ~~deliveries~~ births in our study was similar to that found in most other urban surveys [13 14 20-26] from India *(Table 6).* In the Mumbai slum study[25], the prevalence varied from 6-16% across 48 slum clusters. Tradition was the most important reason behind home ~~delivery~~ births in this study. Apart from the predictors that we identified, poor housing, lack of water supply and hazardous location were associated with home ~~deliveries~~ ~~i~~births in the Mumbai study, indicating that apart from individual and household level factors, the type of neighbourhoods also played a role. One of the limitations of our study is that we could not evaluate cluster level predictors of home ~~delivery~~ births as we included only three clusters in this study.

Migrant status was one of the important determinants of home ~~delivery~~ births in our study. In an ~~A~~analyses using NFHS data, Singh et al[27] reported that urban poor migrants were at highest risk of unsafe ~~delivery~~ birthing practices in contrast to non-poor, non-migrants who were at least risk. In our study sample almost 60% of the households were migrants from neighbouring states, but ~~and~~ most ~~had been~~ living in Delhi for more than 5 years. In spite of

For peer review only - http://bmjopen.bmj.com/site/about/guidelines.xhtml

For peer review only

this, these households were less likely to possess ration, BPL or RSBY cards which are required for availing entitlements to healthcare.

Quality of care did not figure in the discussions as a factor for choosing home births. The quantitative data also confirm that majority of women who visited hospital for ANC and for birthing were quite satisfied with the services offered. We hypothesize that since the focus on facility based birthing and offering free services to enable the same is comparatively new to the urban poor community, the community has not reached the stage of assessing the quality of care and using that as a factor in making a decision on where to deliver. Based on our observation of the facilities there is lot of scope for improvement in the services being offered suggesting a gap between what level of services women perceive they are entitled to, and what they actually receive.

The ~~dissatisfaction~~ discomfort of hospitals expressed by those who gave birth at home ~~among the care seekers that we observed~~ could be attributed to the overburdening of referral hospitals leading to lack of personalized care. Antenatal care is provided at dispensaries, maternal and child care centres (MCH), secondary level and referral hospitals. However, MCH centres cater to deliveries of multigravida only, and all primigravida are referred to the secondary level or referral hospitals leading to increased patient load at these centres. Initiatives to decentralize care to reduce burden on referral hospitals by upgrading the MCH centres has been rather slow. It might be possible that other supply-side issues could have also contributed to this level of dissatisfaction, but due to delay in obtaining permissions, we were unable to conduct facility assessment to identify the potential causes.

Table 6: Prevalence and reasons for home ~~delivery~~ births from urban surveys in India since 2000

Author, year	Study area and target	Sample size and prevalence of	Reasons for home

For peer review only - http://bmjopen.bmj.com/site/about/guidelines.xhtml

of publication (ref)	population/ Study design	home ~~deliveries~~births	~~deliveries~~home births
Rahi et al, 2006[17]	One Urban slum in Delhi, Births recorded during April-June 2005, cross sectional survey	n= 82 births<br><br>Home ~~deliveries~~births= 56.1%	Not reported
Agarwal[18] et al, 2007	One urban slum in Delhi, Women who delivered last 1 year, Cross sectional survey	n= 82<br>Home ~~deliveries~~ births = 31.8%	Lack of awareness for need for check-up (27%)<br>Lack of knowledge about service availability (17%)<br>Long waiting time (22%)<br>None to accompany (15%)<br>Finance (12%)<br>Fear of hospitals (7%)<br>Family objections (2%)
DLHS Fact sheet (2007-8)[9]	Delhi state in 2008 using multistage stratified probability sampling	n= 9689 households<br>Home births<br>Rural = 42.6%<br>Urban = 29.9%<br>Total = 30.8%	Not reported
Thind et al, 2008[15]	NFHS survey data from Maharashtra , cross sectional survey	n=1510 recent births<br>Home ~~deliveries~~births(overall) = 37%<br>Only Urban = 15.3%	Predisposing factors Religion(Hindu), multiple births and caste
Agarwal S et al , 2010[19]	11 slums of Indore, MP, Cross sectional survey of mothers of infants(2004-06)	n= 312<br>Home births~~deliveries~~ = 56.4%	Not reported
Das S, 2011[20]	Mumbai slums from 6 municipal wards, survelllance study (2005-2007)	n= 10,754 births<br>Home ~~deliveries~~ births =10%	Customary(28%) , No time to reach hospital(13%), no body to go along(8%), Fear(7%)
Dasgupta et al, 2006[16]	Rural and urban clusters in West Bengal from Birbhum district. Cross sectional survey, women who delivered in the last one year	n= 320<br>Home ~~deliveries~~ births (rural and urban combined) =51.88%	Not reported
Khan Z et al, 2009 [21]	Periurban area of Aligarh, Uttar Pradesh	n=92 mother of infants<br>Home ~~deliveries~~ births = 60%	Tradition (42%)<br>Related to economics(31%)
Hazarika, 2009[8]	NFHS- 3 Delhi data, Cross sectional survey, women who delivered 6 months ago	n= 2420 (slum dwellers)<br>Home ~~deliveries~~births= 22.62%	Not reported

For peer review only

For peer review only - http://bmjopen.bmj.com/site/about/guidelines.xhtml

Qualitative data from our study suggest that there was a lack of perceived risk among women and their family members. According to the mothers and the family members who played an important role in decision making, the practice of giving birth at home was common and that they had witnessed their relatives doing well after doing the same. Priority was given to other domestic responsibilities and tradition over safe ~~delivery~~births. This may have been particularly relevant among multiparous women.

A low literacy level of the HOH was another important predictor indicating the need to raise awareness of safe birthing practices. The role of CHWs in improving maternal and neonatal health indicators by increasing awareness is well known.[28] Though the Delhi State Health Mission has deployed Accredited Social Health Activists (ASHA) in the urban areas replicating the rural model, a recent evaluation has shown several implementation gaps.[29]

Afsana et al from Bangladesh report that cost, fear of hospitals due to lack of privacy, unfamiliar surroundings and stigma attached to hospital delivery were key reasons for women to choose home births.[30] While women in our study ~~were~~ spoke about fear and embarrassment as deterrents, direct costs associated with delivery and stigma attached to a hospital delivery were not mentioned as factors affecting their choice.~~.~~ Traditionally, Indian women ~~have~~ gave birth~~delivered~~ at home ~~s~~ surrounded by close family members. There is evidence to show that the support of a female relative during the birth process is beneficial and leads to better birth outcomes.[31] The state of Tamil Nadu has implemented a successful birth companion scheme through its public health system which addresses the issues of social support, fear and embarrassment.[32] This model provides a prototype that may be replicated, with suitable context-specific modifications, to address this important barrier to institutional deliveries.

For peer review only

For peer review only - http://bmjopen.bmj.com/site/about/guidelines.xhtml

In our study most births at home were conducted by Dai who lacked professional training in safe birthing practices. An extensive review by Bergstrom and Goodburn has shown that traditional birth attendants had no impact on any reduction in maternal mortality[33]. A meta-analysis of training birth attendants improved survival but the studies included in the review were from high mortality burden rural population and not urban population. [34]Koblinsky et al[6] analysed national level data from several countries which reduced the MMR drastically since 1950 and showed that maternal deaths could be reduced by providing training to *dais or* professionals developing a partnership with *dais*, however it required apart from political will, effective outreach and referral mechanisms that support traditional system of birthing. Experiences from Malaysia and Sri Lanka show that, women are willing to move from home based to facility-based ~~delivery~~ care if transport and services are made free for all, improved awareness and also ensured quality of services at facilities.[6] In India, currently there is no program at national scale for promoting or scaling up community based SBA. The National Rural Health Mission of Government~~t~~ of India has its main strategy for reduction in maternal mortality focused on facility based intra-partum care and provision of -Emergency Obstetrics -Care ~~(EmOC)~~ .

Current initiatives by the government aimed at improving MCH indicators of the urban poor do not directly address some of the key elements identified in our study. Identification and mapping of the most vulnerable populations within the city, sensitization of health professionals to the needs and fears of women, improving reach of ~~U~~CHW to the marginalized, empowering women with information regarding their healthcare entitlements, provision of BPL, ration and RSBY cards to the neediest are some of the key issues that need focused and aggressive implementation.

For peer review only

For peer review only - http://bmjopen.bmj.com/site/about/guidelines.xhtml

It is important for health departments to~~;~~ strengthen the supply side, be more accessible to those who need them the most and establish faith among the community. India needs to explore innovative ways at all levels of care to make birthing~~delivery~~ practices safer. There is hope that the urban health situation will improve in the coming years with the NUHM, if we intervene at the individual, community, system and policy level. The ANCHUL project and similar such endeavours all over the country will feed to provide innovative scalable strategies for the betterment of our urban community.

Acknowledgements: We would like to acknowledge all the families who volunteered to provide data for this survey. We thank the efforts of field staff (Field coordinators, Field supervisor, and Field interviewers), Data manager and Data entry operators.

Contributors~~hip statement~~: SGJ and ND conceived and designed the study with additional inputs from AHS and SZ. ND analysed the quantitative data and prepared the first draft of the manuscript. SMG analysed the qualitative data and wrote the first draft of the qualitative findings. GA, and AHS supervised data collection and commented on drafts of the manuscript. HN and AHS commented on drafts of the manuscript. SZ is the Principal Investigator of ANCHUL, had overall responsibility for ANCHUL, and commented on drafts of the manuscript. All authors contributed to critique and modification of the manuscript, read and approved the final version. ND had full access to all the data in the study and had final responsibility for the decision to submit for publication.

Funding: WHO**,** Geneva (Alliance for Health Policy and Systems Research (HSS/AHPSR), The Special Programme for Research and Training in Tropical Diseases (IER/TDR), The Special

For peer review only - http://bmjopen.bmj.com/site/about/guidelines.xhtml

Programme of Research, Development and Research Training in Human Reproduction (HRP), Child and Adolescent Health and Development (FCH/CAH)) (Grant 2011/139172-0). The funders did not have any role in study design; the collection, analysis, and interpretation of data; the writing of the article or the decision to submit this article for publication

Ethics Approval: The study protocol was approved by Health Ministry Screening Committee of the Government of India, Institutional ethics committees of the Public Health Foundation of India, All India Institute of Medical Sciences, WHO Geneva and Harvard School of Public Health.

Data sharing statement: All unpublished data related to this research project is available with the authors and can be requested by emailing to niveditha@iiphd.org.

For peer review only - http://bmjopen.bmj.com/site/about/guidelines.xhtml

Legends:

Figure 1: Quantitative survey sampling

Figure 2: Conceptual framework of factors leading to home births among urban poor

REFERENCES

1. Ronsmans C, Graham WJ, Lancet Maternal Survival Series steering g. Maternal mortality: who, when, where, and why. Lancet 2006;**368**(9542):1189-200 doi: 10.1016/S0140-6736(06)69380-X[published Online First: Epub Date]|.

2. Montgomery AL, Ram U, Kumar R, et al. Maternal mortality in India: causes and healthcare service use based on a nationally representative survey. PLoS One 2014;**9**(1):e83331 doi: 10.1371/journal.pone.0083331[published Online First: Epub Date]|.

3. Koblinsky M, Matthews Z, Hussein J, et al. Going to scale with professional skilled care. Lancet 2006;**368**(9544):1377-86 doi: 10.1016/S0140-6736(06)69382-3[published Online First: Epub Date]|.

4. Campbell OM, Graham WJ, Lancet Maternal Survival Series steering g. Strategies for reducing maternal mortality: getting on with what works. Lancet 2006;**368**(9543):1284-99 doi: 10.1016/S0140-6736(06)69381-1[published Online First: Epub Date]|.

5. Ronsmans C, Scott S, Qomariyah SN, et al. Professional assistance during birth and maternal mortality in two Indonesian districts. Bull World Health Organ 2009;**87**(6):416-23

6. Koblinsky M, Kureshy N. Safe motherhood case studies: learning with stakeholders in South Asia--an introduction. J Health Popul Nutr 2009;**27**(2):89-92

7. WHO,UNICEF,UNFPA and The World Bank estimates**:** Trends in Maternal Mortality: 1990 to 2010, http://whqlibdoc.who.int/publications/2012/9789241503631_eng.pdf, (Accessed on 30th Decemebr, 2012).

8. Available from http://www.unescap.org/stat/apex/2/apex2_s.2_india_mdgs.pdf (accessed on 1st June, 2012).

9. Women and health: Health service coverage data by country: http://apps.who.int/gho/data/node.main.223, (Accessed December 30th, 2012).

For peer review only - http://bmjopen.bmj.com/site/about/guidelines.xhtml

10. Janani Suraksha Yojna. http://www.mohfw.nic.in/NRHM.htm (accessed on Jan 2013).

11. Lim SS, Dandona L, Hoisington JA, et al. India's Janani Suraksha Yojana, a conditional cash transfer programme to increase births in health facilities: an impact evaluation. Lancet 2010;**375**(9730):2009-23 doi: 10.1016/S0140-6736(10)60744-1[published Online First: Epub Date]|.

12. Randive B, Diwan V, De Costa A. India's Conditional Cash Transfer Programme (the JSY) to Promote Institutional Birth: Is There an Association between Institutional Birth Proportion and Maternal Mortality? PLoS One 2013;**8**(6):e67452 doi: 10.1371/journal.pone.0067452[published Online First: Epub Date]|.

13. Hazarika I. Women's reproductive health in slum populations in India: evidence from NFHS-3. Journal of urban health : bulletin of the New York Academy of Medicine 2010;**87**(2):264-77 doi: 10.1007/s11524-009-9421-0[published Online First: Epub Date]|.

14. District Level Household and Facility Survey-Factsheet, Delhi, Available at http://www.rchiips.org/pdf/rch3/state/Delhi.pdf.

15. National Urban Health Mission, http://www.uhrc.in/module-ContentExpress-display-ceid-95.html (Accessed on Dec 31, 2012).

16. Available at http://pipnrhm-mohfw.nic.in/index_files/non_high_focus_small/Delhi/Executive_Summary_2011-12_final.pdf.

17. NCR policy zones, Demographic profile and Settlement pattern (2001) Available at http://ncrpb.nic.in/pdf_files/SG1.pdf.

18. JJ cluster list; Available from http://delhishelterboard.in/main/. As accessed on 11th July,2012.

19. Vyas S, Kumaranayake L. Constructing socio-economic status indices: how to use principal components analysis. Health policy and planning 2006;**21**(6):459-68 doi: 10.1093/heapol/czl029[published Online First: Epub Date]|.

20. Thind A, Mohani A, Banerjee K, et al. Where to deliver? Analysis of choice of delivery location from a national survey in India. BMC Public Health 2008;**8**:29 doi: 10.1186/1471-2458-8-29[published Online First: Epub Date]|.

21. Dasgupta S, Das P, Mandal NK, et al. A study on intranatal care practices in a district of West Bengal. Indian J Public Health 2006;**50**(1):15-8

For peer review only - http://bmjopen.bmj.com/site/about/guidelines.xhtml

22. Rahi M, Taneja DK, Misra A, et al. Newborn care practices in an urban slum of Delhi. Indian J Med Sci 2006;**60**(12):506-13

23. Agarwal P SM, Garg S. Maternal health care utilization among women in an urban slum in Delhi. Indian Journal of Community Medicine 2007;**32**(3):203-05

24. Agarwal S, Sethi V, Srivastava K, et al. Birth preparedness and complication readiness among slum women in Indore city, India. J Health Popul Nutr 2010;**28**(4):383-91

25. Das S, Bapat U, More NS, et al. Prospective study of determinants and costs of home births in Mumbai slums. BMC Pregnancy Childbirth 2010;**10**:38 doi: 10.1186/1471-2393-10-38[published Online First: Epub Date]|.

26. Khan Z, Mehnaz S, Khalique N, et al. Poor perinatal care practices in urban slums: possible role of social mobilization networks Indian J Community Med 2009;**34**(2):102-07

27. Singh PK, Rai RK, Singh L. Examining the effect of household wealth and migration status on safe delivery care in urban India, 1992-2006. PLoS One 2012;**7**(9):e44901 doi: 10.1371/journal.pone.0044901[published Online First: Epub Date]|.

28. Lassi ZS, Haider BA, Bhutta ZA. Community-based intervention packages for reducing maternal and neonatal morbidity and mortality and improving neonatal outcomes. Cochrane Database Syst Rev 2010(11):CD007754 doi: 10.1002/14651858.CD007754.pub2[published Online First: Epub Date]|.

29. Sixth Common Review Mission Report. Available at http://nhsrcindia.org/pdf_files/resources_thematic/Health_Sector_Overview/NHSRC_Contribution/Final_6thCRM_Book_NRHM.pdf. Accessed on May 2013.

30. Afsana K, Rashid SF. The challenges of meeting rural Bangladeshi women's needs in delivery care. Reprod Health Matters 2001;**9**(18):79-89

31. Hodnett ED, Gates S, Hofmeyr GJ, et al. Continuous support for women during childbirth. Cochrane Database Syst Rev 2012;**10**:CD003766 doi: 10.1002/14651858.CD003766.pub4[published Online First: Epub Date]|.

32. Padmanaban P, Raman PS, Mavalankar DV. Innovations and challenges in reducing maternal mortality in Tamil Nadu, India. J Health Popul Nutr 2009;**27**(2):202-19

33. Bergstrom S GE. The role of traditional birth attendants in the reduction of maternal mortality. Stud Health Serv Organ Policy 2001;**17**:77-96

For peer review only

For peer review only - http://bmjopen.bmj.com/site/about/guidelines.xhtml

34. Wilson A, Gallos ID, Plana N, et al. Effectiveness of strategies incorporating training and support of traditional birth attendants on perinatal and maternal mortality: meta-analysis. BMJ 2011;**343**:d7102 doi: 10.1136/bmj.d7102[published Online First: Epub Date]|.

For peer review only

For peer review only - http://bmjopen.bmj.com/site/about/guidelines.xhtml

Figure 1: Quantitative survey sampling

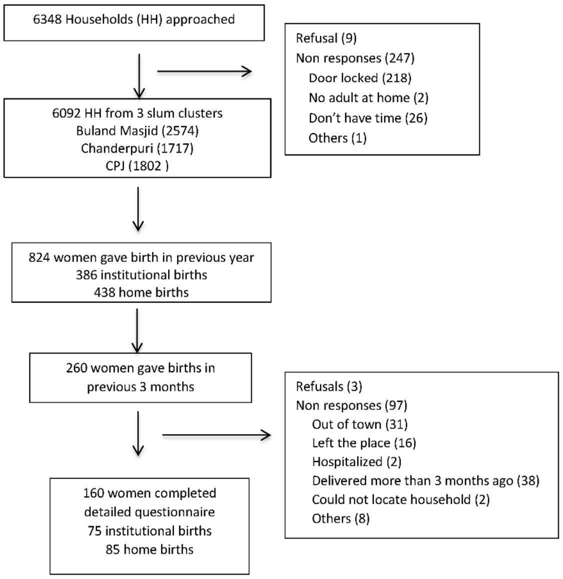

Quantitative survey sampling
210x297mm (300 x 300 DPI)

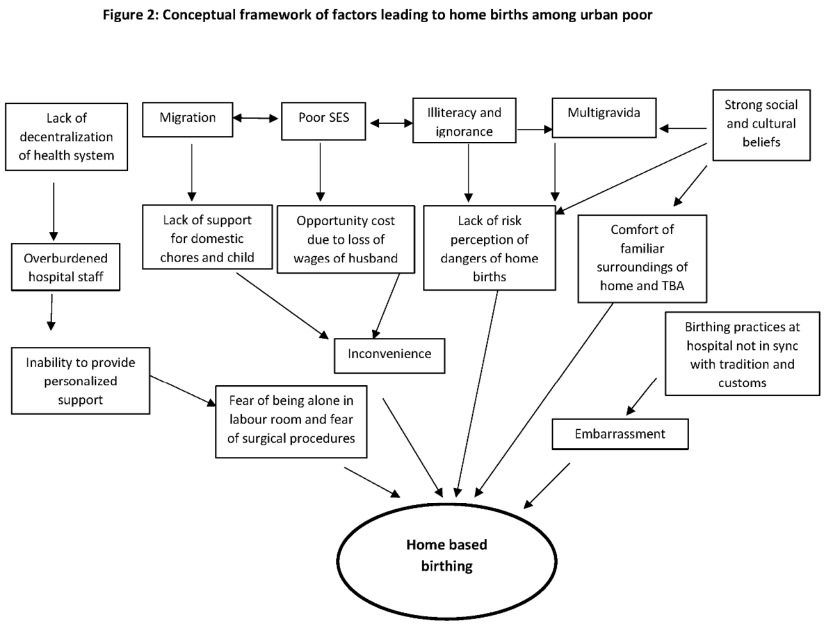

Conceptual framework of factors leading to home births among urban poor
210x297mm (300 x 300 DPI)

STROBE 2007 (v4) Statement—Checklist of items that should be included in reports of *cross-sectional studies*

**Section/Topic**	**Item #**	**Recommendation**	**Reported on page #**
**Title and abstract**	1	(*a*) Indicate the study's design with a commonly used term in the title or the abstract	2
		(*b*) Provide in the abstract an informative and balanced summary of what was done and what was found	2
**Introduction**			
Background/rationale	2	Explain the scientific background and rationale for the investigation being reported	5
Objectives	3	State specific objectives, including any prespecified hypotheses	6
**Methods**			
Study design	4	Present key elements of study design early in the paper	7
Setting	5	Describe the setting, locations, and relevant dates, including periods of recruitment, exposure, follow-up, and data collection	7
Participants	6	(*a*) Give the eligibility criteria, and the sources and methods of selection of participants	8
Variables	7	Clearly define all outcomes, exposures, predictors, potential confounders, and effect modifiers. Give diagnostic criteria, if applicable	9
Data sources/ measurement	8*	For each variable of interest, give sources of data and details of methods of assessment (measurement). Describe comparability of assessment methods if there is more than one group	NA
Bias	9	Describe any efforts to address potential sources of bias	*10*
Study size	10	Explain how the study size was arrived at	8
Quantitative variables	11	Explain how quantitative variables were handled in the analyses. If applicable, describe which groupings were chosen and why	10
Statistical methods	12	(*a*) Describe all statistical methods, including those used to control for confounding	10
		(*b*) Describe any methods used to examine subgroups and interactions	NA
		(*c*) Explain how missing data were addressed	NA
		(*d*) If applicable, describe analytical methods taking account of sampling strategy	10
		(*e*) Describe any sensitivity analyses	NA
**Results**			
Participants	13*	(a) Report numbers of individuals at each stage of study—eg numbers potentially eligible, examined for eligibility, confirmed eligible, included in the study, completing follow-up, and analysed	10
		(b) Give reasons for non-participation at each stage	10
		(c) Consider use of a flow diagram	Figure 1
Descriptive data	14*	(a) Give characteristics of study participants (eg demographic, clinical, social) and information on exposures and potential confounders	11-12
		(b) Indicate number of participants with missing data for each variable of interest	NA
Outcome data	15*	Report numbers of outcome events or summary measures	13-14

For peer review only

Main results	16	(*a*) Give unadjusted estimates and, if applicable, confounder-adjusted estimates and their precision (eg, 95% confidence interval). Make clear which confounders were adjusted for and why they were included	14
		(*b*) Report category boundaries when continuous variables were categorized	14
		(*c*) If relevant, consider translating estimates of relative risk into absolute risk for a meaningful time period	NA
Other analyses	17	Report other analyses done—eg analyses of subgroups and interactions, and sensitivity analyses	NA
**Discussion**			
Key results	18	Summarise key results with reference to study objectives	18
Limitations	19	Discuss limitations of the study, taking into account sources of potential bias or imprecision. Discuss both direction and magnitude of any potential bias	19
Interpretation	20	Give a cautious overall interpretation of results considering objectives, limitations, multiplicity of analyses, results from similar studies, and other relevant evidence	20
Generalisability	21	Discuss the generalisability (external validity) of the study results	20
**Other information**			
Funding	22	Give the source of funding and the role of the funders for the present study and, if applicable, for the original study on which the present article is based	24

*Give information separately for cases and controls in case-control studies and, if applicable, for exposed and unexposed groups in cohort and cross-sectional studies.

Note: An Explanation and Elaboration article discusses each checklist item and gives methodological background and published examples of transparent reporting. The STROBE checklist is best used in conjunction with this article (freely available on the Web sites of PLoS Medicine at http://www.plosmedicine.org/, Annals of Internal Medicine at http://www.annals.org/, and Epidemiology at http://www.epidem.com/). Information on the STROBE Initiative is available at www.strobe-statement.org.

For peer review only
